# Genome-wide association analysis of *Septoria tritici* blotch for adult plant resistance in elite bread wheat (*Triticum aestivum* L) genotypes

**DOI:** 10.1371/journal.pone.0317603

**Published:** 2025-03-10

**Authors:** Molla Mekonnen Kassie, Tiegist Dejene Abebe, Ermias Abate Desta, Wuletaw Tadesse

**Affiliations:** 1 Bahir Dar University, College of Agriculture and Environmental Sciences, Bahir Dar, Ethiopia; 2 Amhara Regional Agricultural Research Institute, Bahir Dar, Ethiopia; 3 Plant Breeding Department, University of Bonn, Bonn, Germany; 4 Sasakawa Africa Association (SAA), Addis Ababa, Ethiopia; 5 International Center for Agricultural Research in the Dry Areas (ICARDA), Rabat, Morocco; Texas A&M University and Texas A&M Agrilife Research, UNITED STATES OF AMERICA

## Abstract

*Septoria tritici* blotch (STB) is a predominant foliar disease of wheat, caused by the pathogen *Zymoseptoria tritici*. This disease can lead to substantial yield losses warranting control by using expensive fungicides. One effective method of STB control is the utilization of resistant wheat varieties. In this particular study, a panel comprising of 186 bread wheat genotypes was assessed for their adult plant resistance (APR) to STB. Field trials were conducted across five environments in Ethiopia during the 2022 and 2023 growing seasons under natural infestation conditions. The association panel was genotyped using 20K single nucleotide polymorphism (SNP) markers. To determine the relationship between genetic markers and STB resistance, a mixed linear model (MLM) analysis was performed using the statgen GWAS R software package. Heritability estimates for STB resistance ranged from 0.39 to 0.95, underscoring the genetic variability and the potential for selection. The study identified 52 marker-trait associations (MTAs) for STB resistance at maturity (SDSM) and 62 MTAs at heading (SDSH). Chromosome 5A contains a high concentration of MTAs that confer resistance to STB, hosting multiple significant MTAs, including four consistently associated markers (‘Kukri_c10033_724’, ‘RAC875_rep_c116420_103’, ‘TG0019’, and ‘RAC875_c30566_230’). Additionally, chromosomes 1B, 2B, 5B, and 7A were found to harbor important MTAs, contributing to resistance across various environments. Notably, two QTLs, qtSTB23 (5A) and qtSTB38 (7B), exhibited stability across multiple environments, making them robust candidates for breeding programs. Furthermore, novel resistance loci on chromosome 2A were discovered, offering new opportunities for enhancing resistance. Therefore, these findings provide an opportunity for improving STB resistance through gene stacking using marker-assisted selection (MAS).

## Introduction

Pests and pathogens cause approximately 21% yield loss in wheat production worldwide [1]. *Septoria tritici* blotch (STB) is a major foliar disease in wheat. The causative agent of STB is an ascomycete fungus *Zymoseptoria tritici (Syn. Mycospharella graminicola;* anamorph *Septoria tritici*). It is a hemibiotrophic fungus that penetrates wheat leaves through the stomata and grows very slowly in the intercellular spaces of the mesophyll cells [2]. After an extended latent period of symptomless growth in the leaf, the symptoms of STB become observable when change in the fungal reproductive mode from asexual to sexual arises [3]. Under favorable environmental condition highly significant yield loss is caused by STB which necessitates the application of fungicides at very high expenses to protect the crop from the menace of the disease [4,5]. In addition to the high cost of fungicides, the frequent use of fungicides leads the rapid emergence of fungicide-resistant strains in different field populations of *Z. tritici* [6–8]. Heavy use of fungicide is not only escalating cost of production but also impacting the environment [9,10]. Evidence on contribution of agricultural pesticides including fungicides to greenhouse gas (GHGE) are also accumulating [11].

Integrated use of host resistance and other agronomic management practices is a sustainable approach to mitigate the impact of STB. Wheat resistance breeding plays a major role in reducing the environmental impact of chemical applications by developing cultivars with enhanced resistance [12]. Thus, characterization of a new source of resistance to STB and introgression of resistance into adapted cultivars to develop high yielding and adapted varieties with high level of disease resistance is an environmentally and socially safe and economical feasible strategy for efficient management of the disease and enhance sustainable agriculture and food security. Use of disease resistant varieties is more important to smallholders’ who cannot afford heavy use of fungicides [13].

Both qualitative and quantitative resistance have been known for STB resistance in wheat. Resistance is controlled by major genes known as qualitative or race-specific resistance, which shows complete resistance and follows a gene-for-gene model [14,15], whereas non-race-specific resistance or quantitative resistance is controlled by a large number of minor genes and has been reported in many wheat cultivars at both the adult and seedling stages [16–18]. To date, 22 major STB resistance genes [19] and 167 quantitative trait locus (QTLs) conversing STB resistance have been identified and mapped [20]. However, the rapid evolution of *Z. tritici* populations owing to sexual reproduction under field conditions has led to the emergence of new virulent strains that can overcome the identified major resistance genes [21]. In contrast, a large number of minors to moderate the effect of minor genes inherited quantitatively are helpful to those of major qualitatively inherited genes. Owing to the collective effect of many genes, quantitative resistance to STB is more durable than qualitative resistance against various *Z. tritici* isolates [12].

The two most well-known methods for determining the genetic architecture of complex traits in crops are genome-wide association studies (GWAS) and linkage mapping (LM) [22]. The development of SNP markers with the advancement in high-throughput genotyping and sequencing technology makes available dense marker coverage across many genomes at a relatively low cost [23]. Most of the Septoria tiriti blotch resistance (Stb) genes were identified using bi-parental QTL mapping, which is important for pyramiding resistance genes. However, the identified allelic differences were restricted to the two parental genotypes of the developed progenies. GWAS can be performed in germplasm collections or natural populations and artificially constructed population, which can provide a wider opportunity to study allelic diversity. The advantages of GWAS compared to QTL mapping include broader allele coverage, increased mapping resolution, and decreased cost and time to establish bi-parental mapping populations [24].

The objective of the present study was to assess a panel of elite bread wheat genotypes composed of 186 breeding lines for APR under natural infestation in Ethiopia, and to determine marker-trait associations for resistance to STB at APR stages using GWAS.

## Materials and methods

In this study, 186 elite bread wheat (*T. aestivum* L.) genotypes obtained from the spring bread wheat breeding program of International Center for Agricultural Research in the Dry Areas (ICARDA) were used. The panel included several prominent wheat genotypes that have been extensively utilized as parents in the ICARDA breeding program. The details of the genotypes are given in Supplementary Table 3 in S1 Appendix.

### Multi-environment trials

The study materials were evaluated under natural infestation conditions during the 2022 and 2023 growing seasons at four locations in Ethiopia. The trial was planted at Adet Agricultural Research Center (11°39’02” Northing, 37°10’81” Easting and an altitude of 2200 m a.s.l) during 2022 and 2023 main cropping seasons, Dabat station (13° 05’ Northing, 37°50’ Easting and an altitude of 2740 m a.s.l) during 2023 main cropping season, Kulumsa Agricultural Research Center (8° 00’54” Northing, 39°09’31” Easting and an altitude of 2217 m a.s.l) during 2023 main cropping season. During the off season (December to April) the experiment was conducted under irrigation at Koga research sub-station (11°20′57.85” Northing and 37°7′29.72” Easting and at an altitude of 1953 m a.s.l.). The experimental design was a partially balanced lattice with two replications, each of which was further divided into 25 rows and ten columns. Each genotype was planted in two rows, with a length of 1 meter and a row-to-row distance of 20 cm, and a plot-to-plot distance of 0.4 meters.. The distance between adjacent plots was 0.4 m. The seed rate was 150 kg/ha, with seeds drilled evenly in rows, and fertilizer was applied according to the recommendations for each specific area. Weeding was performed manually three times during each season.

### *Septoria tritici* blotch evaluation

The Septoria disease severity (SDS) was recorded visually plot-wise by considering the percentage of necrotic leaf area of the four upper most infected leaves using a double-digit scale (00-99) modified from [25] for wheat foliar diseases. The first digit (0–9) represented disease development in terms of plant height (for example, 5 if the disease reaches the middle of the plant height, 8 for reaching the flag leaf, and 9 for reaching the spike), and the second digit stands for severity per se (*e.g.,* 1 for 10% to 9 for 90%). Data were recorded at two time points: heading (SDSH) and maturity stage (SDSM). For each score, the percentage of disease severity was calculated from the 00-99 score using the following formula [26,27]:


$$SDS=\left[\left(\frac{D1}{9}\right)\left(\frac{D2}{9}\right)\right]100$$

Where D1 is the vertical disease progress derived from the average relative height of the disease (0–9). D2 is the severity of the disease, and is measured as the average relative coverage of the diseased leaf area recorded from the top four leaves. SDS values range from zero to 100, where 0 indicates complete resistance and 100 indicates complete susceptibility. Genotypes were classified into the following categories: immune (00), resistance (01–15), moderately resistance (15-35), moderately susceptible (36-55) and susceptible (55-79) and highly susceptible (>79) [28].

## Statistical data analysis

### Phenotypic data analysis

The combined analysis of variance (ANOVA) was conducted for five environment, and three variance components, the environmental variance, genotypic variance and interaction of the genotype and environmental variance were calculated for STB using restricted maximum likelihood estimation procedure using MATA-R version 6.0 software

Broad-sense heritability (H^2^) was calculated using the following Equation:


$$H^{2}=\frac{\sigma_{G}^{2}}{\left[\left(\sigma_{G}^{2}\right)+\left(\frac{\sigma_{G*E}^{2}}{E}\right)+\left(\frac{\sigma_{e}^{2}}{r*E}\right)\right]}$$

Where $\sigma_{G}^{2}$, $\sigma_{G*E}^{2}$ and $\sigma_{e}^{2}$ were the genotypic, genotype ×  environment interaction and error variance, respectively, r represents the number of replications, and E represents the number of environments the trials were conducted.

### Population structure analysis

To know the population structure of elite bread wheat stress panel, a model-based Bayesian cluster analysis was performed using STRUCTURE software (v.2.3.4). The program was run for three replicates for every supposed subpopulation ranking from k = 1 to k = 10 under the admixture model of population structure. Burn-in iteration was 20,000 followed by 20,000 Markov chain Monte Carlo (MCMC) replications after burn-in for each run. To identify the optimum number of sub-populations/clusters the best K value was used. The best K value was obtained as Delta K (ρK) from structure harvester [29] using the log probability of the successive structure repetitions.

### Genotyping and genome-wide association analysis

The spring bread wheat panel was genotyped using 20K SNP array from Trait Genetics containing 23,197 SNP markers. After quality control, 10,613 SNP markers were found to be informative and used for GWAS analysis. Association mapping of Septoria disease traits with genome-wide SNPs was determined by performing a single-trait GWAS following a single-locusmixed linear model (MLM) analysis using the statgen GWAS R software package [30] in the R software [31]. The first ten principal components (PCs) were included as covariate to control population structure, and vanRaden kinship matrix was included to account for hidden relatedness [32]. GWAS was carried out for two Septoria disease traits including SDSH and SDSM on the mean calculated in individual environments, as well as for pooled experimental data and SNPs passing the quality criteria. Those markers with minor allele frequency (MAF) < 0.05, with >  20% missing values per individual genotype, duplicate and markers with unknown positions removed and missing values were imputed using the independent ‘beagle’ method [33] before running GWAS analysis. Quantile-quantile (QQ-plot) generated using–log10 p-value were visually assessed to determine how well the model accounted for population structure and family relatedness between study samples. An arbitrary threshold to declare significant marker-trait associations (MTA) P <  0.001(-log10 [P] >  3.0) was used as described by (Alemu et al. 2021a, 2021b; Maccaferri et al., 2016). StatgenGWAS R was used to visualize the Manhattan and Q Q plots. High-confidence candidate genes from the identified resistance-associated regions were retrieved using the latest IWGSC RefSeq Annotation v2.1, available on (https://wheat-urgi.versailles.inrae.fr/Seq-Repository/Annotations).

## Result

### Phenotypic evaluation

The ANOVA for STB severity at heading and maturity showed a significant difference between genotypes and for the combined and environment-specific analysis (Table 1). The disease severity showed an increasing trend from heading to maturity. The mean Septoria disease severity (SDS) values of each environment at the heading and maturity stages ranged from 3.86–16.38 and 17.22-60.18, respectively, and the highest was recorded at Dabat-2023 (93.37) growing season. The lowest septoria disease severity at maturity (SDSM) value was recorded at Adet-2022 (2.29) (Table 1). Broad-sense heritability for SDSM in each environment ranged from 39.51 at Kulumsa-2023 to 94.69 at Adet-2023 (Table 1).

**Table 1:** Genotypic variance (σ2g) and heritability estimate for SDS traits measured in 186 bread wheat genotypes at five different environments.

Traits		AD-22	AD-23	DA.-23	KO-23	KU-23
SDSH	Min	0.85	1.23	2.68	2.11	2.8
	Max	28.74	9.88	69.84	17.3	11.26
	Mean	6.07	3.86	16.38	5.99	4.71
	VarG	16.796***	2.179***	74.188***	5.468***	5.505***
	VarR	6.4639	2.6341	43.7866	4.8535	16.85
	H2	0.8386	0.6233	0.7721	0.6926	0.3951
SDSM	Min	2.29	13.04	20.44	5.71	7.85
	Max	88.82	78.49	93.37	61.03	29.28
	Mean	45.32	43.85	60.18	31.00	17.22
	VarG	616.73***	251.454***	177.309***	113.026***	29.43***
	VarR	69.258	28.1769	193.334	75.107	16.58
	H^2^	0.9468	0.9469	0.6471	0.7506	0.3951

Traits: SDSH, Septoria disease severity at heading; SDSM, Septoria disease severity at maturity; Environment: AD-22, Adet 2022; AD-23, Adet-2023; D.A.-23, Dabat-2023; KO-23, Koga-2023; KU-23, Kulumsa-2023. Variance: genetic variance (VarG), environmental variance (VarE); error variance (VarR). H^2^, broad-sense heritability; *** Significant at P < 0.001.

The combined ANOVA showed that the effects of genotype, year, location, and their two-way interaction (genotype ×  location) were significant for SDS traits (Table 3). The combined environment broad-sense heritability was high (0.65) for SDSM and lower (0.51) for SDSH (Table 2). The SDSM for the STB values of 186 genotypes followed a normal distribution, ranging from 15.39–58.92 with a standard deviation and mean value of 9.9 to 41.41, respectively. Similarly, the Septoria disease severity at heading (SDSH) values of the genotypes followed a normal distribution ranging from 2.06 to 20.55 with standard deviation and mean value of 3.11 and 7.9, respectively.

**Table 2:** Variance components and Heritability of SDS traits of 186 wheat genotypes based on pooled data from five environments.

Combined						
Traits	Mean	H^2^	Var G	Var G * E	VarE	VarR
SDSH	7.4	0.51	4.91***	15.88***	25.28**	14.96
SDSM	39.52	0.65	74.86***	162.08***	249.73**	81.57

Traits: SDSH, Septoria disease severity at heading; SDSM, Septoria disease severity at maturity; Variance: VarG, genetic variance, VarG * E, genotype-by-environment interaction variance; VarE, environmental variance; VarR, error variance. H2, broad-sense heritability; *** Significant at P < 0.001.

**Table 3:** Combined ANOVA for SDS traits.

Source of variation	DF	Mean Square	
		SDSH	SDSM
Genotypes	186	108.5***	1273***
Location	3	14777.7***	57239***
Genotype*location	558	57.7***	454***
Location*Replication	4	30.3^NS^	4568***
Location*replication*block	72	39.7***	223***

*** Significant at P < 0.001, ^NS^ Non-significant.

**Fig 1:**
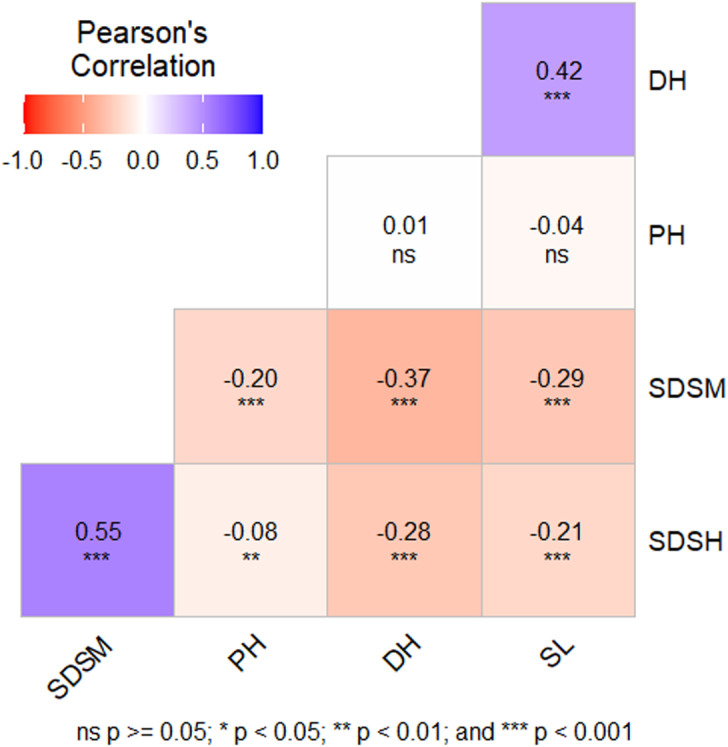
The correlation between disease severity parameters and agronomic traits evaluated, with correlation coefficients reported for each phenotype pair. The intensity of the correlation is indicated by shading according to the color bar above. * , ** and *** indicate significances, at p < 0.05, p < 0.01and p < 0.001, respectively and ns indicate p > 0.05.

Disease resistance traits were correlated with certain agronomic traits measured in the same experimental field (Fig 1). The plant’s phenology is associated with disease progression, evidenced by a significant negative correlation between SDS and days to heading. The analysis revealed notable negative correlations between septoria disease severity at maturity and days to heading (-0.20), spike length (-0.29), and plant height (-0.37).

## Population structure analysis

**Fig 2:**
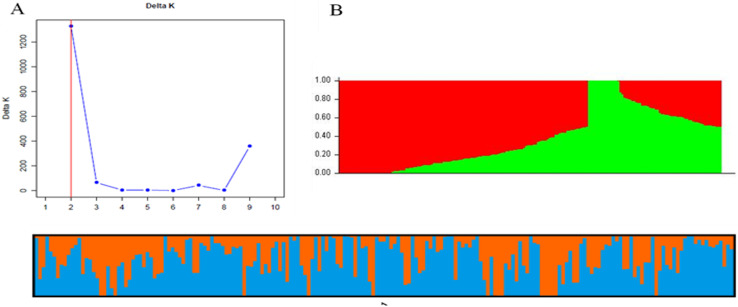
Population structure of 186 bread wheat genotypes. A) Best delta K value estimated using. Evanno et al. (2005) method, and the pick at k = 2 shows the number of sub population in the panel. (B) Population structure of sub-population one and sub-population.

**Fig 3:**
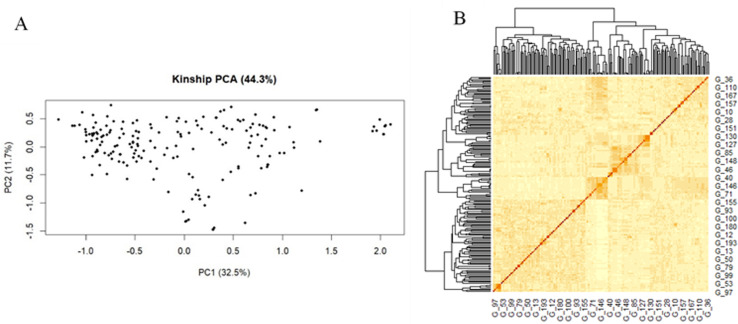
Principal component and familiar relatedness analysis of 186 wheat genotypes. A) PCA plots of the first two principal components to depict the samples’ relationship in space, and (B) Kinship displayed through heat map and a tree.

Two sub-populations were identified in the association panel via STRUCTURE analysis (Fig 2). Approximately 64.5% (120) of the genotypes were assigned to cluster one and 35.5% (66) of the genotypes were grouped into cluster two. A Q-Q plot of the two sub-populations in the association panel is displayed in Fig 2B. The Clumpark result detected a greater degree of admixture among the two sub-populations (Fig 2C). Most of the tested individual genotypes shared alleles inherited from both subgroups, confirming the presence of close relationships among the study genotypes. The ∆K technique was used to analyze the population structure of 186 genotypes of bread wheat. Two distinct groups in the tested genotypes were found using the PCA-based population structure analysis (Fig 3A) and the ∆K technique (Fig 2A, B).

The kinship principal component analysis (PCA) also suggested that the presence of two sub-populations the first PCA explained 44.3% (PC1 = 32.5% and PC2 = 11.7%) of the total variances contained in the data (Fig 3A). The analysis also confirmed the presence of kinship in the association panel (Fig 3B), it suggested that the use of powerful statistical GWAS model that accounts for the familial relatedness and population structure in the association study.

## Evaluation of marker distribution

**Fig 4:**
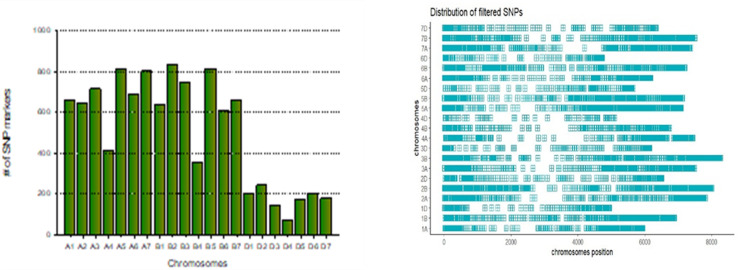
The SNP marker distribution on 21 chromosomes of wheat: A, B and D the three sub-genomes.

From an initial set of 23,197 SNP markers on the 20k SNP array, a total of 10,613 markers were retained after filtering. The filtering process eliminated markers with a minor allele frequency below 0.05, those with more than 20% missing data, duplicate SNPs, and markers lacking known positions. The distribution of the mapped markers over the wheat genomes showed that the D sub-genome contained the lowest number of markers, whereas the B genome contained the largest number of markers. In total, 4738 SNP markers were distributed in the A sub-genome, 4656 in the B sub-genome, and 1219 in the D sub-genome. Chromosome-wide distribution analysis showed the highest marker density on chromosome 2 B (835), followed by 5 B (814) and 5A (813). In contrast, 4D (73), 3D (146), and 5D (175) had the lowest number of markers (Fig 4).

### Linkage disequilibrium and genome-wide association analysis

**Fig 5:**
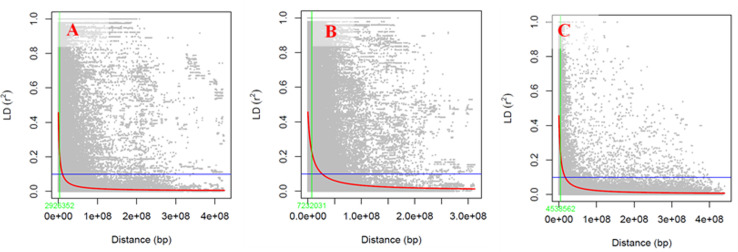
Linkage disequilibrium decay for genome A (A), genome B (B), genome D (C) of 186 bread wheat genotypes.

In this research, the degree of linkage disequilibrium (LD) was assessed for the A, B, and D sub-genomes of wheat. The B sub-genome displayed the highest level of linkage, with an average r^2^ value of 0.22. This was followed by the A and D sub-genomes, which had r^2^ values of 0.18 and 0.14, respectively. The A sub-genome showed the fastest LD decay, observed at 2,926,352 base pairs (bp), while the D sub-genome demonstrated a slower LD decay at 4,538,562 bp (Fig 5). The B sub-genome had the slowest LD decay among the three, occurring at 7,232,031 bp. These results highlight distinct genetic linkage and decay patterns across the various wheat sub-genomes.

Genome-wide association analysis was performed to identify genomic regions associated with resistance to STB. Fast single-trait GWAS following the methods described in [34], which is one of a series of statistical genetic packages for streamlining the analysis of typical breeding experiments developed by biometris, was used in this study. The GWAS was accompanied by SDS data combined over all locations, and then by SDS data collected at each location separately. We report and discuss MTA with an arbitrary threshold of P <  0.001(-log10 [P] >  3.0).

**Fig 6:**
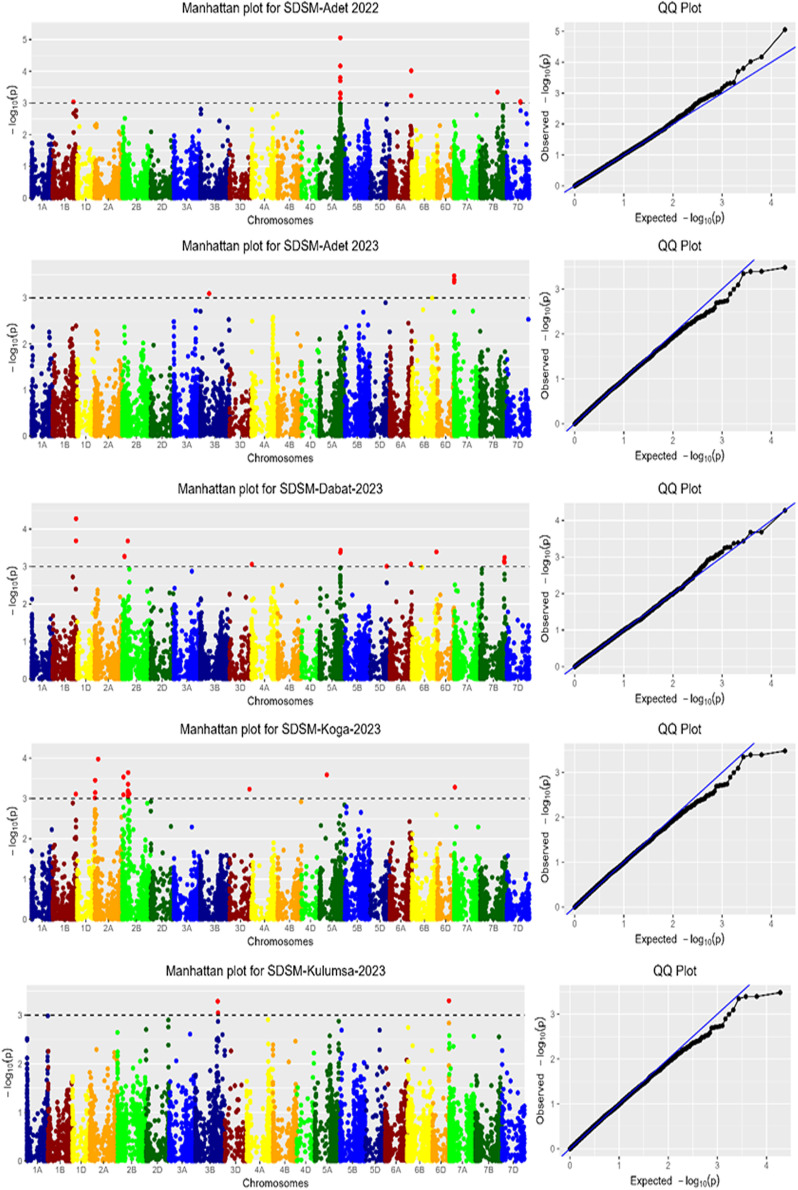
Manhattan plots for SDSM data measured in five locations. Each dot represents a SNP. The y-axis is the –log10 of the p-value depending the significance of the association test. X-axis is the genomic position of the SNPs on corresponding chromosome. The QQ plot shows how well the GWAS model accounted for population structure and kinship for SDSM.

**Markers associated with SDSM:** The genome-wide association (GWA) analysis identified 52 marker-trait associations (MTAs) linked to resistance against *Septoria tritici* blotch (STB) at the maturity stage (SDSM), explaining 6.17% to 15.59% of the phenotypic variation. These MTAs were distributed across multiple environments and chromosomes. At Koga, 17 MTAs were identified on chromosomes 1B, 2A, 2B, 3D, 5A, and 7A, while 14 MTAs associated with general SDSM resistance were mapped to chromosomes 1B, 2B, 4A, 5A, 5D, 6A, 6B, and 7B. Thirteen MTAs were located on chromosomes 1B, 5A, 6A, 7B, and 7D at Adet in 2022, and five were identified on chromosomes 3B, 6D, and 7A at Adet in 2023. Similarly, Kulumsa in 2023 revealed three MTAs on chromosomes 3B, 6D, and 7A (Fig 6). Across all environments, the MTAs were distributed as follows: 11 on chromosome 2B (21.15%), 10 on 5A (19.23%), five each on 2A and 7A (9.62%), four each on 1B and 7B (7.69%), three each on 3B and 6A (5.77%), two on 7D (3.85%), and one each on 3D, 4A, 5D, 6B, and 6D (1.92%). This analysis highlights the significance of chromosomes 2B and 5A, which account for over 40% of the identified MTAs, providing valuable targets for breeding programs aimed at enhancing STB resistance at the maturity stage (Appendix Table 1).

**Fig 7:**
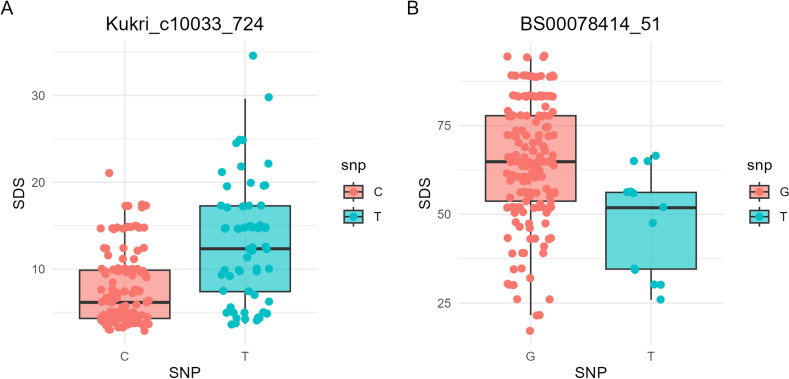
Boxplot showing resistance values for SDS 186 bread wheat genotypes. A: Marker Kukri_c10033_724 carrying either the resistance (C) or susceptible (T) allele at maturity at Dabat-2023, B: Marker BS00078414_51 carrying either the resistance (T) or susceptible (G) allele at heading at Adet 2023.

The marker Kukri_c10033_724 on chromosome 5A was identified as the most significant at Adet in 2023, with a marker effect of 2.4 on the SDS value at heading, explaining 7.93% of the genetic variance and achieving a LOD score of 5.56. Genotypes carrying the “C” allele of this SNP predominantly exhibited resistance to SDS at heading. Similarly, the marker BS00078414_51 on chromosome 1B was highlighted as a significant marker at Dabat, with a marker effect of –10.96 on the SDS value at maturity. This marker explained 10.84% of the genetic variance and had a LOD score of 4.27. In contrast, genotypes possessing the “G” allele of this SNP were generally susceptible to SDS at maturity, as shown in Fig 7.

**Fig 8:**
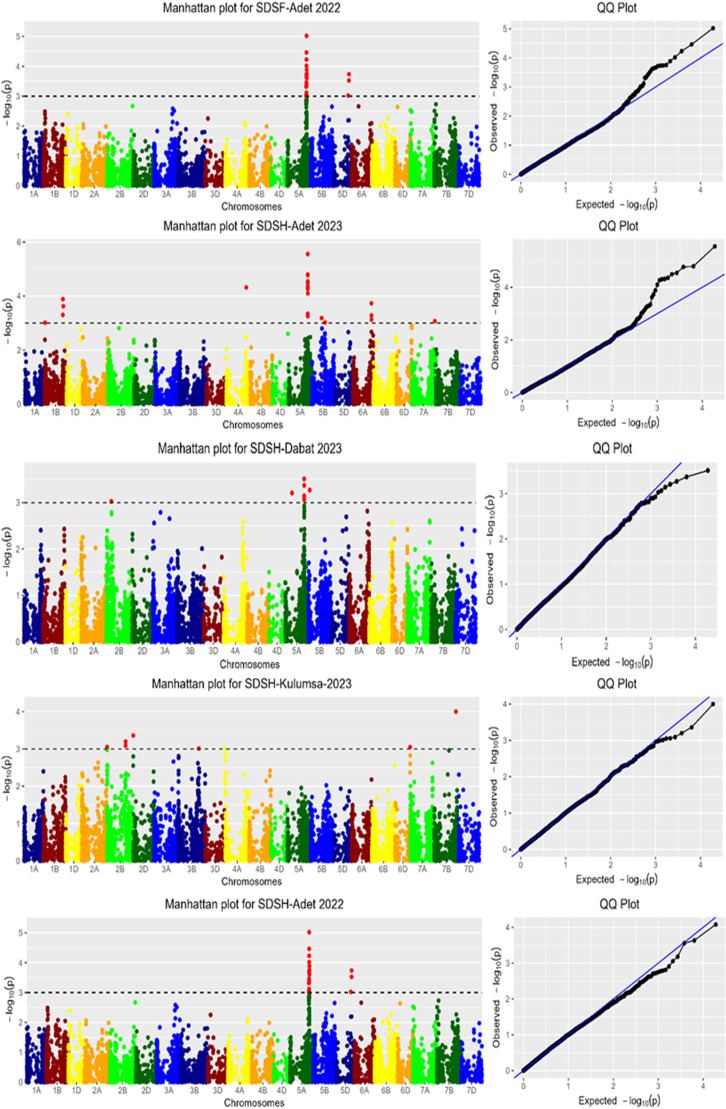
Manhattan plots for SDSH data measures in five locations.

**Markers associated with SDSH:** The genome-wide association scan (GWAS) for SDSH identified 62 marker-trait associations (MTAs) linked to resistance against *Septoria tritici* blotch (STB) at the heading stage (Appendix Table 2), with individual markers explaining 6.35% to 15.06% of the phenotypic variance. At Adet in 2023, 22 MTAs were identified on chromosomes 1B, 4A, 5A, 5B, 6A, 7A, and 7D, while 21 MTAs were detected at Adet in 2022 on chromosomes 5A and 5B. In Dabat and Kulumsa, 7 MTAs were located on chromosomes 2B, 2D, 3B, 5A, 5B, and 7A, and at Koga, 5 MTAs were identified on chromosomes 2A, 4D, 5B, and 7B (Fig 8). Across all environments, the MTAs were distributed as follows: 35 (55.56%) on chromosome 5A, 5 (7.93%) on chromosome 5B, 4 (6.35%) each on chromosomes 1A and 1B, 3 (4.76%) each on chromosomes 5D and 6A, 2 (3.17%) on chromosome 7B, and 1 (1.59%) each on chromosomes 2A, 2D, 3B, 4A, 4D, 6D, and 7A. This highlights chromosome 5A as a major contributor to STB resistance at the heading stage (Appendix Table 2).

Out of the 114 MTAs identified, fifteen SNP markers were significantly associated with STB resistance across multiple locations, with a notable concentration on chromosome 5A. Four SNP markers: Kukri_c10033_724, RAC875_rep_c116420_103, TG0019, and RAC875_c30566_230 on chromosome 5A were consistently associated with significant allelic effects on STB mean values across three environments (Adet-2022, Adet-2023, and Dabat-2023). Seven additional SNP markers AX-158538950, IAAV1650, Nsnp_Ex_rep_c66689_65010988, Nsnp_Ex_c31799_40545376, tplb0038h19_1394, RAC875_c13931_205, and Nsnp_Ex_c2702_5013188 on chromosome 5A were associated with resistance in two environments (Adet-2022 and Adet-2023). Two SNP markers Excalibur_c41710_417 (5D) and Nsnp_Ex_c55777_58153636 (5A) were significantly associated with resistance in Adet-2022 and Dabat-2023, while two additional markers AX-95148023 (5A) and Ra_c4397_542 (2B) were associated with resistance in Koga-2023 and Dabat-2023. There were no any common marker between Kulumsa and other environments observed.

Potential quantitative trait loci (QTL) for *Septoria tritici* resistance were identified by grouping marker-trait associations (MTA) based on their genomic locations, using a physical distance window (in Mbp) determined through genome-wide SNP pairwise linkage disequilibrium (LD) analysis. The genome-wide pairwise LD (r2) values between SNPs on each chromosome, plotted against their physical distance, are illustrated in Figure 5. MTAs located within the chromosome-specific LD decay distance on the same linkage group were considered part of the same potential QTL. Based on these LD criteria, a total of 39 potential QTLs were identified, incorporating 114 MTAs (Table 4).

**Table 4:** Summary of the putative QTLs identified across bread wheat chromosomes for STB resistance.

Potential QTL	Chr	Map position (bp)	No. of MTAs	Phenotype Environment
qtSTB.01	1B	21473796	1	Adet 2023 H
qtSTB.02	1B	563254727 - 598339967	4	Adet 2022 M, Adet 2023 H,
qtSTB.03	1B	649005218 - 669948745	3	Dabat 2023 M, Koga 2023 M
qtSTB.04	2A	4620089-21095554	4	Koga 2023 M
qtSTB.05	2A	102685812	1	Koga 2023 M
qtSTB.06	2A	719566734	1	Koga 2023 H
qtSTB.07	2B	1832830	1	Kulumsa 2023 H
qtSTB.08	2B	28138249-42619355	2	Koga 2023 M
qtSTB.09	2B	57876972-72456453	2	Dabat 2023 M
qtSTB.10	2B	150464672 - 175028367	7	Koga 2023 M, Dabat 2023 M, Dabat 2023 H
qtSTB.11	2B	200385458	1	Koga 2023 M
qtSTB.12	2B	565080256-579821341	2	Kulumsa 2023 H
qtSTB.13	2D	8297177	1	Kulumsa 2023 H
qtSTB.14	3B	252595780	1	Adet 2023 M
qtSTB.15	3B	623076245 - 644321527	3	Kulumsa 2023 M, Kulumsa 2023 H
qtSTB.16	3D	563861601	1	Koga 2023 M
qtSTB.17	4A	17265696	1	Dabat 2023 M
qtSTB.18	4A	616874298	1	Adet 2023 H
qtSTB.19	4D	403572723	1	Koga 2023 H
qtSTB.20	5A	205132950	2	Dabat 2023 H, Koga 2023 M
qtSTB.21	5A	570714642	1	Adet 2022H
qtSTB.22	5A	577942781	1	Adet 2022 H
qtSTB.23	5A	581620919 - 597890151	41	Adet 2022 M, Adet 2022 H, Adet 2023 H, Dabat 2023 H, Dabat 2023 M
qtSTB.24	5B	50469684	1	Dabat 2023 H
qtSTB.25	5B	307408948	1	Adet 2023 H
qtSTB.26	5B	409876449	1	Adet 2023 H
qtSTB.27	5B	424925845 - 441798211	2	Koga 2023 H,
qtSTB.28	5D	451674901	1	Adet 2022 M
qtSTB.29	5D	470473562 - 480049293	2	Dabat 2023 M, Adet 2022 H
qtSTB.30	6A	560812338 - 567805780	3	Adet 2023 H
qtSTB.31	6A	594818372	1	Dabat 2023 M
qtSTB.32	6A	598636889 - 605651331	2	Adet 2022 M
qtSTB.33	6D	463743278	2	Kulumsa 2023 H, Kulumsa 2023 M
qtSTB.34	7A	977272 - 8132084	4	Adet 2023 M
qtSTB.35	7A	22834490	1	Koga 2023 M
qtSTB.36	7A	704792521	1	Adet 2023 H
qtSTB.37	7B	491397671	1	Adet 2022 M
qtSTB.38	7B	658449259 - 709463743	5	Dabat 2023 M, Koga 2023 H, Kulumsa 2023 H
qtSTB.39	7D	386646259	2	Adet 2022 M

H and M after the year indicates heading and Maturity stages where the marker show significant association

Out of the 39 potential QTLs identified, two QTLs, qtSTB 23 and qtSTB 38, were stable across three environments. qtSTB 23, localized on chromosome 5A, was consistently detected at Adet 2022 (both at heading and maturity), Dabat 2023 (both at heading and maturity), and Adet 2023 (at heading). Similarly, qtSTB 38, localized on chromosome 7B, was stable across Dabat 2023 (at maturity), Koga 2023, and Kulumsa 2023 (both at heading). In addition, five QTLs exhibited stability across five environments: qtSTB 2, localized on chromosome 1B, at Adet 2022 (maturity) and Adet 2023 (heading); qtSTB 3, localized on chromosome 1B, at Dabat 2023 and Koga 2023 (both at maturity); qtSTB 10, localized on chromosome 2B, at Koga 2023 (maturity) and Dabat 2023 (at both heading and maturity); qtSTB 20, localized on chromosome 5A, at Dabat 2023 (heading) and Koga 2023 (maturity); and qtSTB 29, localized on chromosome 5D, at Dabat 2023 (maturity) and Koga 2022 (heading). These stable QTLs represent promising targets for validation and incorporation into breeding programs aimed at enhancing wheat resistance to *Septoria tritici*.

The functional association of the identified potential QTLs for STB resistance was further investigated by annotating genes found in the QTL regions using the recently released IWGSC_v2.1. This analysis revealed several genes associated with disease resistance and plant defense. For example, the high-confidence gene (HCG) TraesCS2A03G0029000, located on chromosome 2A near qtSTB04, encodes a Knottin scorpion toxin-like domain-containing protein. Similarly, HCG TraesCS2A02G014100.1, located near qtSTB04, encodes a protein involved in rRNA N-glycosylase activity. On chromosome 1B, near qtSTB03, the HCG TraesCS1B02G424700.1 encodes Auxin Response Factors (ARFs), which are transcription factors. Additionally, the HCG TraesCS1B03G1166400, located near qtSTB03, encodes a Mitogen-activated protein kinase. On chromosome 4A, near qtSTB18, the HCG TraesCS4A03G0831700 encodes an RIN4 pathogenic type III effector avirulence factor, containing an Avr cleavage site domain. High-confidence gene on chromosome 3B, near qtSTB.15, the gene TraesCS3B03G0975100 encodes an Osmotin-Like Protein, defense tool against biotic and abiotic stresses [35].

## Discussion

New sources of resistance against wheat diseases, including STB, are needed to ensure high yield and acceptable grain quality of wheat. Various studies have been conducted to identify new QTL for STB resistance [3,12,27,36–42].

This study evaluated 186 genetically diverse elite bread wheat (*T. aestivum* L.) genotypes obtained from the spring bread wheat breeding program of ICARDA to detect QTLs for adult plant STB disease resistance. We found a strong genotype x environment interaction in addition to a wide range of genetic variability in the STB disease resistance response in adult plants, which is usually due to unequal disease pressure across locations. High broad-sense heritability within the individual environments (H^2^ = 64.71–94.69%) suggested that the variation is heritable. This is crucial because high heritability indicates a robust selection response when combined with significant and large genetic variation. High heritability (H^2^ = 74%) for STB resistance has been reported for elite spring bread wheat lines of ICARDA in Morocco [39] and (H^2^ = 89%) for CIMMYT genotypes and Ethiopian commercial cultivars in Ethiopia [41]. The study also showed that location, genotype and all interaction effects affect STB infestation and confirms the importance of germplasm evaluation across multiple locations and over years at hot spots to find stable and durable STB-resistant genotypes.

The correlation analysis revealed significant negative correlations between septoria disease severity at maturity and days to heading, indicating that genotypes with shorter plant height and earlier flowering are more susceptible to *Zymoseptoria tritici* infection. This result aligns with Simón et al. (2005) [43], who found a negative correlation between shortness and septoria disease resistance, and Gerard et al. (2017) [44], who similarly reported consistently significant negative regression coefficients relating heading date to STB resistance.

STRUCTURE and principal component analysis revealed the presence of population stratification. Two sub-populations were suggested by the analysis of the population. The MLM sufficiently accounted for population stratification, marker effects, and familial relatedness, and as a result, reduced the confounding effects that could lead to false-positive MTAs. This was confirmed by visualizing the Q-Q and Manhattan plots. Similar population stratification and weak population sub-structuring have been reported for 180 bread wheat genotypes in Ethiopia [41].

The SNP marker distribution among the wheat genomes was unequal; the D genome contained a relatively small number of SNPs (1219), and the A (4738) and B (4656) genomes contained a relatively large number of SNPs. This unequal SNP distribution has also been reported in different studies of bread wheat genotypes [39,41,45]. The distribution of SNP in the wheat sub-genome can be influenced by multiple factors: the genetic complexity of hexaploid wheat and the size of the genome may contribute to an uneven distribution of SNP. The distinct sub-genomes of wheat possess varying evolutionary histories, which can result in differential levels of SNP density and genetic diversity.

The GWAS analysis identified 52 marker-trait association for SDSM and 63 MTAs for SDSH traits where markers had *p* <  0.001 (-log10 [p] > 3.0). The number of SDS MTAs identified in our study is higher than the report of [27] who reported 35 significant associations for Ethiopian durum wheat panel and almost equivalent to the findings of Mekonnen et al. (2021) who reported 53 MTAs for STB resistance in Ethiopia.

The 2022 and 2023 growing seasons in Dabat and Adet were characterized by prolonged rainfall and high relative humidity, which created favorable conditions for the development of Septoria diseases. In Adet 2022, these conditions led to the highest natural incidence of *Septoria tritici* blotch, with 34 MTA identified, including 13 attributed to SDSM and 21 to SDSH. Similarly, 28 MTA were recorded in Adet 2023, and 21 MTA in Dabat 2023, underscoring the significant impact of these environmental factors on Septoria disease Severity. The presence of heavy rainfall leads to water splashing in the wheat field, which favors the spread of pycnidia, resulting in high levels of STB in susceptible genotypes. The progression of STB in wheat is influenced by environmental factors such as relative humidity, air temperature, rainfall, leaf wetness duration and inoculum concentration [5,46,47]. Across these three environments, four markers showed common MTA, highlighting potential targets for further genetic studies.

Chromosome 5A contains a high concentration of genes that confer resistance to STB. This genomic region encompasses a large number of significant markers. Chromosome 5AL has been widely recognized as a significant region associated with resistance to *Septoria tritici* blotch. The stb17 gene, reported by Tabib Ghaffary et al. (2012) [48] in adult plants of synthetic hexaploid wheat, was mapped at 62 cM. Similarly, Muqaddasi et al. (2019) identified multiple QTNs associated with STB resistance on chromosome 5A, spanning 48.1–90.4 cM, in adult European winter wheat plants. Dreisigacker et al. (2015) also identified a QTL between 29.4–36.9 cM on chromosome arm 5AL linked to adult plant resistance (APR) to STB in spring bread wheat. Alemu et al. (2021a) reported significant marker-trait associations in the 63 cM region of chromosome 5A, while Mekonnen et al. (2021) identified a related map location at 685.96 Mbp in Ethiopian wheat germplasm. Collectively, these findings underscore the critical role of chromosome 5A in the genetic architecture of STB resistance across diverse wheat genotypes and environments. Chromosome 5B also contains large concentration of MTA that confer resistance of STB. One *Stb* namely genes *Stb* 1 [50] were reported in this genomic region. Similarly significant MTA were reported on chromosome 5B by (Mekonnen et al., 2021). Similarly, chromosome 1A and 1B are the genomic regions having large number of MTA in our study. The genomic region 1B is known for the source of *Stb 11* [51] and significant MTA was also reported in this genomic region by [3,41,42,52].

The discovery of these associations provides a valuable resource for future breeding programs aimed at improving STB resistance in wheat, potentially contributing to the development of more resilient wheat varieties.

The identification of 39 potential QTLs for *Septoria tritici* resistance, covering 114 MTAs, highlights the genetic complexity of resistance to *Zymoseptoria tritici*. Grouping MTAs based on genomic locations and pairwise LD analysis allowed the delineation of QTLs, providing a robust framework for understanding the genetic basis of resistance [53]. Among these, two QTLs, qtSTB 23 on chromosome 5A and qtSTB 38 on chromosome 7B, established stability across three environments, suggesting their potential as reliable targets for breeding programs. Additionally, five QTLs (qtSTB 2, qtSTB 3, qtSTB 10, qtSTB 20, and qtSTB 29) exhibited stability across five environments, further reinforcing their potential importance. Notably, qtSTB 23 on chromosome 5A aligns with previously reported resistance loci for Z. tritici [3,49], supporting its significance in STB resistance. The findings of this study provide valuable insights for breeding programs where stable QTLs can be integrated into marker-assisted selection (MAS) strategies to develop STB-resistant wheat genotypes. The consistency of these QTLs across diverse environments underscores their utility in addressing the challenges posed by Z. tritici, a major pathogen in global wheat production systems.

The identification of defense-related candidate genes, such as TraesCS2A03G0029000, TraesCS2A02G014100.1, TraesCS1B02G424700, TraesCS1B03G1166400, TraesCS4A03G0831700, and TraesCS3B03G0975100, near significant markers highlights the potential functional association of these QTL regions with the plant defense system against pathogen infections. For instance, the translations of the genes found near qtSTB04 (TraesCS2A03G0029000) on chromosome 2A and qtSTB18 (TraesCS4A03G0831700) on chromosome 4A are associated with proteins involved in plant immunity. The protein encoded by TraesCS2A03G0029000 contains a Knottin scorpion toxin-like domain, which inhibits fungal growth by disrupting cell membranes or interfering with pathogenic enzymes [35]. Also, the protein encoded by TraesCS4A03G0831700 includes a RIN4 pathogenic type III effector avirulence factor domain, which plays a pivotal role in plant immunity by acting as a hub for effector recognition and resistance protein activation [54]. Additionally, the gene TraesCS1B03G1166400, located near qtSTB03, encodes a Mitogen-activated protein kinase (MAPK). MAPKs are a class of serine/threonine protein kinases that play a central role in regulating cellular processes such as stress responses, growth, development, and immune signaling in plants. These kinases are crucial components of the plant defense mechanism during pathogen attacks [55].

This study identifies a novel potential QTL for *Septoria tritici* blotch resistance, specifically qtSTB.04, qtSTB.05, and qtSTB.06 on chromosome 2A. To the authors’ knowledge, no major STB resistance genes have been previously mapped to this region in wheat, suggesting that these QTLs are likely novel.

## Conclusions

The present study aimed to investigate the genetic architecture of adult-plant resistance to *Septoria tritici* blotch (STB) in bread wheat genotypes using SNP markers and multi-environment phenotypic data. The research findings indicated that the association panel exhibited a significant number of STB resistance alleles that have the potential to be leveraged for enhancing wheat resistance against the predominant *Zymoseptoria tritici* populations in Ethiopia. In addition, the study conducted a genome-wide association study (GWAS) and identified 52 significantly associated markers (MTAs) for septoria disease severity at maturity (SDSM) and 63 MTAs for Septoria disease severity at heading (SDSH).

Notably, 15 SNP markers were consistently associated with STB resistance across multiple locations, with a remarkable concentration on chromosome 5A. Four key SNP markers ‘Kukri_c10033_724’, ‘RAC875_rep_c116420_103’, ‘TG0019’, and ‘RAC875_c30566_230’ on chromosome 5A showed significant allelic effects on STB resistance across three environments: Adet-2022, Adet-2023, and Dabat-2023. Many identified MTAs overlapped with chromosomal locations of previously reported resistance genes, reinforcing their relevance.

Additionally, this study identified novel MTAs for STB resistance on chromosome 2A, to the authors’ knowledge a region where no previously published major STB resistance genes have been mapped, suggesting these MTAs are likely novel. However, the validation of these MTAs is essential before their integration into marker-assisted selection (MAS) programs.

This study emphasizes the potential use of identified MTAs and stable resistant wheat genotypes to develop *Z. tritici*-resistant wheat genotypes in breeding programs. These findings provide valuable insights for facilitating the selection and development of improved wheat varieties with enhanced resistance against STB, contributing to the sustainable management of this economically devastating disease.

## Supporting information

S1 Appendix List of Appendix tables. (DOCX)

S1 Data Phenotypic for SDS traits of 186 genotypes for five tested locations. (CSV)

S2 Data Genotypic data of 186 genotypes. (TXT)

## Abbreviations

ANOVA: Analysis of variance
APR: Adult plant resistance
BLUE: Best linear Unbiased Estimates
GWAS: Genome-wide association studies
ICARDA: International Center for Agricultural Research in the Dry Areas
LM: Linkage mapping
MCMC: Markov chain Monte Carlo
MLM: Mixed linear model
MTA: Marker-trait associations
PC: Principal Components
PCA: Principal Component analysis
QTL: Quantitative trait locus
SDS: Septoria disease severity
SDSH: Septoria disease severity at heading
SDSM: Septoria disease severity at maturity
SNP: Single nucleotide polymorphism
STB: Septoria tritici blotch
