# Supplementary material for: Genome-wide association analysis of Septoria tritici blotch for adult plant resistance in elite bread wheat (Triticum aestivum L) genotypes
Source: PLoS One. 2025 Mar 10;20(3):e0317603. doi: 10.1371/journal.pone.0317603 (PMC11892845; doi:10.1371/journal.pone.0317603)
Supplement: S1 Appendix — (DOCX) [file pone.0317603.s001.docx]

Appendix table 1: Genome-wide association significant marker for SDSM during 2022 at Adet and 2023 at Adet, Koga, Dabat and Kulumsa locations in Ethiopia

| Snp | chromosome | Position (Mb) | allFreq | p. value | effect | Effect Se | RLR^2^ | LOD | %PVE |
| --- | --- | --- | --- | --- | --- | --- | --- | --- | --- |
| **Adet 2022** | | | | | | | | | |
| BS00010436_51 | 1B | 591109967 | 0.34946 | 0.00092 | 7.19889 | 2.1967 | 0.0561 | 3.0345 | 8.62 |
| Kukri_c10033_724 | 5A | 584677768 | 0.32796 | 0.00048 | 7.98659 | 2.3160 | 0.0619 | 3.3186 | 10.29 |
| AX-158538950 | 5A | 585235173 | 0.35484 | 0.00071 | 7.33741 | 2.1914 | 0.0585 | 3.1507 | 9.02 |
| Nsnp_Ex_c31799_40545376 | 5A | 585403320 | 0.33333 | 0.00020 | 8.38989 | 2.2887 | 0.0697 | 3.7006 | 11.45 |
| tplb0038h19_1394 | 5A | 585431093 | 0.32796 | 0.00016 | 8.57582 | 2.3042 | 0.0718 | 3.8029 | 11.86 |
| Nsnp_Ex_rep_c66689_65010988 | 5A | 585609287 | 0.32258 | 0.00007 | 9.05441 | 2.3126 | 0.0791 | 4.1690 | 13.11 |
| TG0019 | 5A | 587423597 | 0.33871 | 0.00001 | 9.75251 | 2.2447 | 0.0965 | 5.0541 | 15.59 |
| Excalibur_c26671_57 | 5A | 591319170 | 0.38710 | 0.00051 | 7.13463 | 2.0784 | 0.0614 | 3.2921 | 8.84 |
| AX-158588027 | 6A | 601566889 | 0.27419 | 0.00059 | 7.12709 | 2.0989 | 0.0601 | 3.2297 | 7.40 |
| AX-158552197 | 6A | 602721331 | 0.30108 | 0.00010 | 7.98561 | 2.0816 | 0.0761 | 4.0174 | 9.82 |
| BS00065626_51 | 7B | 491397671 | 0.40323 | 0.00045 | 7.48870 | 2.1624 | 0.0624 | 3.3438 | 9.88 |
| IACX7714 | 7D | 386646259 | 0.33333 | 0.00090 | -6.41170 | 1.9526 | 0.0563 | 3.0451 | 6.69 |
| IAAV4133 | 7D | 391576333 | 0.34409 | 0.00096 | -6.40471 | 1.9598 | 0.0558 | 3.0197 | 6.77 |
| **Adet 2023** | | | | | | | | | |
| AX-94943216 | 3B | 252595780 | 0.05914 | 0.00081 | 7.35787 | 2.22081 | 0.05731 | 3.09300 | 7.07 |
| Tdurum_contig52015_1320 | 7A | 3907272 | 0.41398 | 0.00033 | -3.89470 | 1.09971 | 0.06521 | 3.47943 | 8.64 |
| BS00095826_51 | 7A | 3983443 | 0.30645 | 0.00046 | -4.06573 | 1.17445 | 0.06240 | 3.34157 | 8.25 |
| Kukri_c2101_2358 | 7A | 4896912 | 0.46237 | 0.00041 | -3.64127 | 1.04292 | 0.06344 | 3.39238 | 7.74 |
| RAC875_c54764_319 | 7A | 5202084 | 0.46774 | 0.00041 | -3.65673 | 1.04767 | 0.06340 | 3.39049 | 7.81 |
| **Dabat 2023** | | | | | | | | | |
| Tdurum_contig57153_1356 | 1B | 661517174 | 0.06989 | 0.00021 | -9.74585 | 2.66527 | 0.06936 | 3.68401 | 8.57 |
| BS00078414_51 | 1B | 662718745 | 0.06989 | 0.00005 | -10.96140 | 2.76166 | 0.08121 | 4.27453 | 10.84 |
| RAC875_c61801_299 | 2B | 65106972 | 0.06989 | 0.00054 | -9.01853 | 2.63690 | 0.06095 | 3.27076 | 7.34 |
| AX-158557650 | 2B | 65226453 | 0.06452 | 0.00055 | -9.44453 | 2.76471 | 0.06081 | 3.26395 | 7.47 |
| Ra_c4397_542 | 2B | 157694672 | 0.20968 | 0.00021 | -6.57757 | 1.80054 | 0.06924 | 3.67768 | 9.95 |
| Nsnp_Ex_c4752_8482625 | 4A | 17265696 | 0.10215 | 0.00087 | -7.06160 | 2.14502 | 0.05660 | 3.05868 | 6.35 |
| TG0019 | 5A | 587423597 | 0.33871 | 0.00043 | 5.60922 | 1.61228 | 0.06300 | 3.37111 | 9.78 |
| Nsnp_Ex_c55777_58153636 | 5A | 594576868 | 0.35484 | 0.00037 | 5.40534 | 1.53745 | 0.06430 | 3.43450 | 9.29 |
| Excalibur_c41710_417 | 5D | 475013562 | 0.36022 | 0.00099 | 5.00599 | 1.53652 | 0.05547 | 3.00358 | 8.02 |
| AX-158526903 | 6A | 594818372 | 0.05376 | 0.00087 | -9.57973 | 2.90754 | 0.05669 | 3.06308 | 6.48 |
| RAC875_c95948_614 | 6B | 697923021 | 0.17204 | 0.00041 | -6.27345 | 1.79775 | 0.06337 | 3.38926 | 7.78 |
| AX-158554057 | 7B | 677114372 | 0.46774 | 0.00072 | -4.95588 | 1.48262 | 0.05830 | 3.14147 | 8.49 |
| RAC875_c40569_716 | 7B | 687673150 | 0.35484 | 0.00078 | -5.08094 | 1.52956 | 0.05760 | 3.10723 | 8.20 |
| AX-109726130 | 7B | 689354982 | 0.36022 | 0.00057 | -5.15315 | 1.51443 | 0.06035 | 3.24140 | 8.50 |
| **Koga 2023** | | | | | | | | | |
| tplb0034p10_1134 | 1B | 656235218 | 0.06452 | 0.00078 | 7.38094 | 2.22206 | 0.05759 | 3.10694 | 7.79 |
| Kukri_c31776_1621 | 2A | 7550089 | 0.36022 | 0.00097 | -3.83106 | 1.17356 | 0.05568 | 3.01399 | 8.01 |
| Kukri_c23195_266 | 2A | 12327530 | 0.34946 | 0.00097 | -3.92006 | 1.20083 | 0.05568 | 3.01398 | 8.28 |
| RAC875_c47161_100 | 2A | 16741486 | 0.35484 | 0.00036 | -4.23995 | 1.20308 | 0.06460 | 3.44923 | 9.75 |
| AX-95177447 | 2A | 18165554 | 0.34946 | 0.00071 | -4.09063 | 1.22258 | 0.05841 | 3.14685 | 9.01 |
| AX-95247247 | 2A | 102685812 | 0.11828 | 0.00011 | -5.92419 | 1.55288 | 0.07527 | 3.97681 | 8.67 |
| RAC875_rep_c113106_93 | 2B | 35368249 | 0.34946 | 0.00081 | -3.97588 | 1.20057 | 0.05726 | 3.09057 | 8.51 |
| AX-94739692 | 2B | 35389355 | 0.35484 | 0.00029 | -4.29588 | 1.20282 | 0.06628 | 3.53200 | 10.01 |
| Ra_c4397_542 | 2B | 157694672 | 0.20968 | 0.00070 | -4.49436 | 1.34047 | 0.05865 | 3.15826 | 7.93 |
| AX-158575304 | 2B | 159888514 | 0.09140 | 0.00087 | -5.59839 | 1.70023 | 0.05662 | 3.05971 | 6.17 |
| Nsnp_JD_rep_c64505_41132927 | 2B | 165111241 | 0.13978 | 0.00065 | -4.97685 | 1.47653 | 0.05925 | 3.18781 | 7.06 |
| AX-158547341 | 2B | 165116471 | 0.14516 | 0.00044 | -5.09822 | 1.46958 | 0.06266 | 3.35414 | 7.64 |
| BS00031814_51 | 2B | 167798367 | 0.16129 | 0.00023 | -5.15774 | 1.41956 | 0.06851 | 3.64209 | 8.53 |
| RFL_Contig5290_1493 | 2B | 200385458 | 0.34409 | 0.00077 | -4.22861 | 1.27157 | 0.05772 | 3.11325 | 9.56 |
| AX-158573025 | 3D | 563861601 | 0.38172 | 0.00059 | -3.82404 | 1.12580 | 0.06015 | 3.23141 | 8.18 |
| AX-95148023 | 5A | 205132950 | 0.33333 | 0.00026 | -4.23683 | 1.17588 | 0.06742 | 3.58801 | 9.45 |
| Kukri_c32980_552 | 7A | 22834490 | 0.37097 | 0.00053 | -3.52705 | 1.02981 | 0.06112 | 3.27893 | 6.88 |
| **Kulumsa 2023** | | | | | | | | | |
| Nsnp_JD_rep_c49010_33257826 | 3B | 630306245 | 0.06452 | 0.00052 | -4.05329 | 1.18211 | 0.06125 | 3.28548 | 7.57 |
| Nsnp_Ex_rep_c108114_91468537 | 3B | 637091527 | 0.15054 | 0.00089 | 2.74044 | 0.83400 | 0.05640 | 3.04865 | 7.33 |
| D_contig10996_530 | 6D | 463743278 | 0.08065 | 0.00051 | -3.58483 | 1.04345 | 0.06149 | 3.29683 | 7.27 |
| SNP: SNP name, chromosome: chromosome on which the SNP is located, Position: position of the SNP on the Chromosome, allFreq: allele frequency of the SNP, p. value: P-value for the SNP, effect: effect of the SNP on the trait value, effect se: standard error of the effect of the SNP on the trait value, RLR^2^: likelihood-ratio-based R^2^, LOD: LOD score for the SNP, defined as–log10(p-Value), %PVE: percent of phenotypic variation accounted | | | | | | | | | |

Appendix table 2: Genome-wide association significant marker for SDSH during 2022 at Adet and 2023 at Adet, Koga, Dabat and Kulumsa locations in Ethiopia

| snp | chromosome | Position (Mb) | allFreq | p. value | effect | Effect Se | RLR^2^ | LOD | %PVE |
| --- | --- | --- | --- | --- | --- | --- | --- | --- | --- |
| **Adet 2022** | | | | | | | | | |
| AX-94406443 | 5A | 570714642 | 0.43011 | 0.00096 | 1.64173 | 0.50264 | 0.05574 | 3.01679 | 8.03 |
| Ku_c19858_2078 | 5A | 577942781 | 0.40860 | 0.00048 | 1.76378 | 0.51154 | 0.06192 | 3.31791 | 9.13 |
| AX-158558692 | 5A | 584550919 | 0.40860 | 0.00076 | 1.74641 | 0.52472 | 0.05782 | 3.11780 | 8.96 |
| IAAV1650 | 5A | 584614233 | 0.34946 | 0.00040 | 1.76946 | 0.50636 | 0.06354 | 3.39768 | 8.65 |
| Kukri_c10033_724 | 5A | 584677768 | 0.32796 | 0.00019 | 1.92617 | 0.52304 | 0.07032 | 3.73132 | 9.94 |
| RAC875_c30566_230 | 5A | 585020739 | 0.34409 | 0.00035 | 1.83853 | 0.52075 | 0.06482 | 3.46014 | 9.27 |
| RAC875_c13931_205 | 5A | 585068163 | 0.34946 | 0.00047 | 1.78243 | 0.51613 | 0.06211 | 3.32731 | 8.78 |
| Nsnp_Ex_c2702_5013188 | 5A | 585068425 | 0.37097 | 0.00091 | 1.64374 | 0.50090 | 0.05625 | 3.04163 | 7.66 |
| RAC875_rep_c116420_103 | 5A | 585068741 | 0.33871 | 0.00023 | 1.90095 | 0.52293 | 0.06858 | 3.64539 | 9.83 |
| AX-158538950 | 5A | 585235173 | 0.35484 | 0.00022 | 1.82331 | 0.50009 | 0.06897 | 3.66477 | 9.25 |
| Nsnp_Ex_c31799_40545376 | 5A | 585403320 | 0.33333 | 0.00010 | 1.98228 | 0.51651 | 0.07613 | 4.02013 | 10.61 |
| tplb0038h19_1394 | 5A | 585431093 | 0.32796 | 0.00006 | 2.04914 | 0.51918 | 0.08034 | 4.23078 | 11.24 |
| Nsnp_Ex_rep_c66689_65010988 | 5A | 585609287 | 0.32258 | 0.00003 | 2.11743 | 0.52110 | 0.08494 | 4.46285 | 11.90 |
| TG0019 | 5A | 587423597 | 0.33871 | 0.00001 | 2.20593 | 0.50944 | 0.09589 | 5.02236 | 13.24 |
| Excalibur_c26671_57 | 5A | 591319170 | 0.38710 | 0.00019 | 1.76125 | 0.47914 | 0.07007 | 3.71892 | 8.94 |
| Nsnp_Ex_c55777_58153636 | 5A | 594576868 | 0.35484 | 0.00013 | 1.84804 | 0.49057 | 0.07346 | 3.88701 | 9.50 |
| AX-158542785 | 5A | 594955146 | 0.36022 | 0.00018 | 1.80647 | 0.48890 | 0.07077 | 3.75372 | 9.14 |
| BS00041063_51 | 5A | 594960151 | 0.36559 | 0.00025 | 1.76320 | 0.48775 | 0.06785 | 3.60912 | 8.76 |
| IAAV9053 | 5D | 451674901 | 0.39785 | 0.00095 | 1.69924 | 0.51961 | 0.05588 | 3.02330 | 8.40 |
| Excalibur_c41710_417 | 5D | 475013562 | 0.36022 | 0.00018 | 1.80435 | 0.48942 | 0.07047 | 3.73869 | 9.12 |
| Tdurum_contig10843_745 | 5D | 475509293 | 0.36022 | 0.00030 | 1.74768 | 0.48985 | 0.06615 | 3.52540 | 8.55 |
| **Adet 2023** | | | | | | | | | |
| AX-111603368 | 1B | 21473796 | 0.25806 | 0.00097 | 1.66937 | 0.51162 | 0.05563 | 3.01149 | 6.35 |
| Excalibur_c45119_251 | 1B | 570484727 | 0.09140 | 0.00050 | 2.64089 | 0.76799 | 0.06159 | 3.30217 | 7.09 |
| Tdurum_contig13117_1316 | 1B | 573571896 | 0.25269 | 0.00013 | 2.23896 | 0.59469 | 0.07338 | 3.88283 | 6.35 |
| RAC875_c10083_154 | 1B | 584148708 | 0.11828 | 0.00024 | 2.50784 | 0.69227 | 0.06813 | 3.62291 | 6.84 |
| BS00064269_51 | 4A | 616874298 | 0.44086 | 0.00005 | 1.92959 | 0.48352 | 0.08206 | 4.31724 | 7.44 |
| IAAV1650 | 5A | 584614233 | 0.34946 | 0.00005 | 2.01982 | 0.50679 | 0.08186 | 4.30696 | 7.92 |
| Kukri_c10033_724 | 5A | 584677768 | 0.32796 | 0.00000 | 2.39640 | 0.52458 | 0.10613 | 5.55609 | 7.93 |
| RAC875_c30566_230 | 5A | 585020739 | 0.34409 | 0.00003 | 2.13299 | 0.52284 | 0.08559 | 4.49573 | 7.35 |
| RAC875_c13931_205 | 5A | 585068163 | 0.34946 | 0.00004 | 2.07848 | 0.51791 | 0.08295 | 4.36197 | 6.89 |
| Nsnp_Ex_c2702_5013188 | 5A | 585068425 | 0.37097 | 0.00045 | 1.73725 | 0.50159 | 0.06246 | 3.34439 | 8.38 |
| RAC875_rep_c116420_103 | 5A | 585068741 | 0.33871 | 0.00002 | 2.21281 | 0.52511 | 0.09106 | 4.77394 | 7.80 |
| AX-158538950 | 5A | 585235173 | 0.35484 | 0.00056 | 1.70616 | 0.50043 | 0.06058 | 3.25264 | 8.30 |
| Nsnp_Ex_c31799_40545376 | 5A | 585403320 | 0.33333 | 0.00005 | 2.05454 | 0.51801 | 0.08110 | 4.26884 | 11.26 |
| tplb0038h19_1394 | 5A | 585431093 | 0.32796 | 0.00003 | 2.13694 | 0.52078 | 0.08655 | 4.54406 | 10.44 |
| Nsnp_Ex_rep_c66689_65010988 | 5A | 585609287 | 0.32258 | 0.00002 | 2.20828 | 0.52268 | 0.09151 | 4.79696 | 11.16 |
| TG0019 | 5A | 587423597 | 0.33871 | 0.00008 | 1.97884 | 0.51032 | 0.07766 | 4.09626 | 11.03 |
| Nsnp_Ex_c16499_25005415 | 5B | 307408948 | 0.23656 | 0.00065 | 1.91993 | 0.57000 | 0.05918 | 3.18398 | 10.92 |
| AX-158599772 | 5B | 409876449 | 0.27957 | 0.00095 | 1.71988 | 0.52612 | 0.05583 | 3.02126 | 11.68 |
| RAC875_c104548_369 | 6A | 563742338 | 0.47849 | 0.00019 | 1.67248 | 0.45420 | 0.07030 | 3.73055 | 12.22 |
| BS00042195_51 | 6A | 564723668 | 0.48925 | 0.00075 | 1.51689 | 0.45512 | 0.05798 | 3.12548 | 11.97 |
| AX-110988092 | 6A | 564875780 | 0.48387 | 0.00053 | 1.57255 | 0.45908 | 0.06114 | 3.27972 | 13.05 |
| RAC875_c66200_288 | 7A | 704792521 | 0.08065 | 0.00085 | 2.68204 | 0.81280 | 0.05686 | 3.07117 | 12.68 |
| **Dabat 2023** | | | | | | | | | |
| Ra_c4397_542 | 2B | 157694672 | 0.20968 | 0.00095 | -3.49437 | 1.06867 | 0.05586 | 3.02262 | 7.59 |
| AX-95148023 | 5A | 205132950 | 0.33333 | 0.00063 | -3.16411 | 0.93626 | 0.05956 | 3.20259 | 8.35 |
| Kukri_c10033_724 | 5A | 584677768 | 0.32796 | 0.00031 | 3.51101 | 0.98663 | 0.06582 | 3.50930 | 10.19 |
| RAC875_c30566_230 | 5A | 585020739 | 0.34409 | 0.00072 | 3.29681 | 0.98647 | 0.05828 | 3.14046 | 9.20 |
| RAC875_rep_c116420_103 | 5A | 585068741 | 0.33871 | 0.00086 | 3.26806 | 0.99162 | 0.05672 | 3.06450 | 8.97 |
| TG0019 | 5A | 587423597 | 0.33871 | 0.00043 | 3.32721 | 0.95693 | 0.06293 | 3.36747 | 9.30 |
| RAC875_c16827_292 | 5B | 50469684 | 0.25269 | 0.00054 | 3.37156 | 0.98566 | 0.06097 | 3.27159 | 8.05 |
| **Koga 2023** | | | | | | | | | |
| AX-94461119 | 2A | 719566734 | 0.17742 | 0.00066 | 1.14843 | 0.34097 | 0.05917 | 3.18359 | 7.26 |
| AX-158537173 | 4D | 403572723 | 0.27419 | 0.00089 | -0.96251 | 0.29275 | 0.05646 | 3.05170 | 6.96 |
| Excalibur_c32630_104 | 5B | 432155845 | 0.29032 | 0.00008 | 1.17233 | 0.30310 | 0.07728 | 4.07736 | 10.68 |
| AX-158525375 | 5B | 434568211 | 0.24731 | 0.00028 | 1.17231 | 0.32705 | 0.06675 | 3.55496 | 9.65 |
| BobNhite_c28058_232 | 7B | 702233743 | 0.07527 | 0.00023 | 1.74102 | 0.47981 | 0.06834 | 3.63348 | 7.96 |
| **Kulumsa 2023** | | | | | | | | | |
| BS00084668_51 | 2B | 1832830 | 0.18280 | 0.00088 | -2.28197 | 0.69344 | 0.05656 | 3.05654 | 7.49 |
| AX-94391786 | 2B | 572310256 | 0.45699 | 0.00063 | -2.10093 | 0.62217 | 0.05946 | 3.19799 | 10.54 |
| Excalibur_rep_c66577_159 | 2B | 572591341 | 0.48925 | 0.00081 | 2.13540 | 0.64483 | 0.05725 | 3.09040 | 10.97 |
| IACX8446 | 2D | 8297177 | 0.15591 | 0.00044 | -2.62629 | 0.75627 | 0.06278 | 3.36019 | 8.74 |
| Nsnp_JD_rep_c49010_33257826 | 3B | 630306245 | 0.06452 | 0.00098 | -3.41672 | 1.04729 | 0.05562 | 3.01070 | 6.78 |
| D_contig10996_530 | 6D | 463743278 | 0.08065 | 0.00089 | -3.03872 | 0.92445 | 0.05644 | 3.05051 | 6.59 |
| Excalibur_c25630_537 | 7B | 665679259 | 0.28495 | 0.00010 | 2.28357 | 0.59659 | 0.07575 | 4.00085 | 10.22 |
| SNP: SNP name, chromosome: chromosome on which the SNP is located, Position: position of the SNP on the Chromosome, allFreq: allele frequency of the SNP, p. value: P-value for the SNP, effect: effect of the SNP on the trait value, effect se: standard error of the effect of the SNP on the trait value, RLR^2^: likelihood-ratio-based R^2^, LOD: LOD score for the SNP, defined as–log10(p-Value) | | | | | | | | | |

Appendix table 3 List of bread wheat genotypes from spring bread wheat breeding program of ICARDA used in the experiment

| G–code | Var | Name/Pedigree | Selection History |
| --- | --- | --- | --- |
| G-1 | 35373 | 02W50807/RSMF8 704//MACE | ISBW14S-85 -0SD-010KUL-1KUL-0KUL |
| G-2 | 33014 | ACHTAR*3//KANZ/KS85-8-4/3/KATILA-17/4/MON'S'/ALD'S'//ALDAN'S'/IAS58/5/HUBARA-1/GOUMRIA-8/6/HIDDAB/ATTILA-7 | ICARC-WIP13050-0TR-0TR-5TR |
| G-3 | 33931 | ACHTAR*3//KANZ/KS85-8-4/3/KATILA-17/4/MON'S'/ALD'S'//ALDAN'S'/IAS58/5/HUBARA-1/GOUMRIA-8/6/HIDDAB/ATTILA-7 | ICARC-WIP13050-020KUL-030DZ-1TR |
| G-4 | 35286 | DUCULA/KAUZ//WEAVER/4/KRONSTAD/3/FRET2/KUKUNA//FRET2 | ISBW14S-10 -0SD-010KUL-3KUL-0KUL |
| G-5 | 34276 | FLORKWA-2/6/SAKER'S'/5/RBS/ANZA/3/KVZ/HYS//YMH/TOB/4/BOW'S'/7/DAJAJ-6/8/SAKHA-94 | ICARC-WIP12124-13KUL-05DZ-5TR |
| G-6 | 34273 | FLORKWA-2/6/SAKER'S'/5/RBS/ANZA/3/KVZ/HYS//YMH/TOB/4/BOW'S'/7/DAJAJ-6/8/SAKHA-94 | ICARC-WIP12124-13KUL-05DZ-2TR |
| G-7 | 33116 | GEMMIZA-12/4/ATTILA 50Y//ATTILA/BCN/3/STAR*3/MUSK-3 | ICARC-WIP13205-0TR-0TR-5TR |
| G-8 | 34016 | GIRWILL-13/2*PASTOR-2//GEMMIZA-9/3/MISR-1 | ICARC-WIP13259-020KUL-030DZ-8TR |
| G-9 | 32332 | GOUMRIA-3//MILAN/MUNIA/3/MILAN/KAUZ//HD29/2*WEAVER | ICW 11-20052-3AP-0TR-2TR |
| G-10 | 32907 | HUBARA-1/3/MUNIA/CHTO//MILAN/4/GOUMRIA-8/5/WARDA | ISBW13-449-0TR-0TR-5TR |
| G-11 | 34502 | HUBARA-15/ZEMAMRA-8//Mace | ISBW14-721-161 |
| G-12 | 34048 | HUBARA-16/2*SOMAMA-3//HUBARA-3*2/SHUHA-4 | ICARC-WIP13316-020KUL-030DZ-5TR |
| G-13 | 32335 | HUBARA-16/6/ALD/CEP75630//CEP75234/PT7219/3/BUC/BJY/4/CBRD/5/TNMU/PF85487/7/OASIS/SKAUZ//4*BCN/3/2*PASTOR | ICW 11-20053-5AP-0TR-1TR |
| G-14 | 32887 | HUBARA-3*2/SHUHA-4//AKBAR | ISBW13-423-0TR-0TR-1TR |
| G-15 | 32835 | HUBARA-3*2/SHUHA-4//BASHIR | ISBW13-305-0TR-0TR-2TR |
| G-16 | 32837 | HUBARA-3*2/SHUHA-4//BASHIR | ISBW13-305-0TR-0TR-4TR |
| G-17 | 32804 | HUBARA-3*2/SHUHA-4//BERKUME | ISBW13-288-0TR-0TR-1TR |
| G-18 | 33817 | HUBARA-3*2/SHUHA-4//HIDDAB/FLORKWA-2 | ISBW13-304-020KUL-030DZ-3TR |
| G-19 | 32843 | HUBARA-3*2/SHUHA-4//HUBARA-3*2/SHUHA-4 | ISBW13-309-0TR-0TR-1TR |
| G-20 | 32799 | HUBARA-3*2/SHUHA-4//KABEER | ISBW13-284-0TR-0TR-2TR |
| G-21 | 32793 | HUBARA-3*2/SHUHA-4//MURAJ | ISBW13-282-0TR-0TR-5TR |
| G-22 | 32809 | HUBARA-3*2/SHUHA-4//NADIA-1 | ISBW13-292-0TR-0TR-2TR |
| G-23 | 32894 | HUBARA-3*2/SHUHA-4//NARC 2011 | ISBW13-427-0TR-0TR-3TR |
| G-24 | 32798 | HUBARA-3*2/SHUHA-4//TOROS | ISBW13-283-0TR-0TR-6TR |
| G-25 | 33805 | HUBARA-3*2/SHUHA-4//TOROS | ISBW13-297-020KUL-030DZ-1TR |
| G-26 | 33776 | HUBARA-3*2/SHUHA-4/4/URES/BOW//OPATA/3/GIRWILL-7 | ISBW13-267-020KUL-030DZ-5TR |
| G-27 | 33536 | HUBARA-3/SHUHA-4//INQALAB 91/FLAG-2 | ICW 11-01002-0TR-0TR-3TR |
| G-28 | 33530 | HUBARA-3/SHUHA-4//REYNA-21 | ICW 11-00997-0TR-0TR-3TR |
| G-29 | 32660 | HUBARA-5/PASTOR-2/3/SHI#4414/CROWS"//GK SAGVARI/CA80DEBEIRA DEBEIRA | ISBW13-067-0TR-0TR-2TR |
| G-30 | 33713 | HUBARA-5/PASTOR-2/6/88ZHONG218//CTK/VEE/3/KVZ/GV//PR/4/KRASNOVODOPADSKAYA25/5/KS82117/MLT | ISBW13-065-020KUL-030DZ-5TR |
| G-31 | 32617 | HUBARA-5/PASTOR-5//ZANDER-17/SIDS-DEBEIRA | ISBW13-004-0TR-0TR-1TR |
| G-32 | 33899 | HUBARA-8/3/MUNIA/ALTAR 84//MILAN/4/ANGI-2/5/BOH4/7/WA476/3/391//NUM/5/W22/5/ANA/6/TAM200/KASYAN | ISBW13-708-020KUL-030DZ-3TR |
| G-33 | 33338 | KAUZ//ALTAR 84/AOS/3/MILAN/KAUZ/4/HUITES/5/KIRITATI/6/KAUZ//ALTAR 84/AOS/3/MILAN/KAUZ/4/HUITES/7/CROC-1/AE.SQUARROSA (224)//OPATA/3/QAFZAH-21/4/SOMAMA-3 | ICW 11-00597-0TR-0TR-3TR |
| G-34 | 33844 | KAUZ//TRAP#1/BOW/3/QAFZAH-21/4/MILAN/SHA7*2//KAUZ | ISBW13-388-020KUL-030DZ-4TR |
| G-35 | 32675 | MELLAL-1/OUEDZEM-1//HUBARA-3*2/SHUHA-4 | ISBW13-114-0TR-0TR-1TR |
| G-36 | 33724 | MELLAL-1/OUEDZEM-1//JOUDI | ISBW13-121-020KUL-030DZ-4TR |
| G-37 | 35524 | MISKEET-18/3/CBRD/WBLL1//PANDION/5/ALD/COC//URES/3/DUCULA/4/METSO | ISBW14T-139 -0SD-010KUL-3KUL-0KUL |
| G-38 | 32724 | MUNIA//CHEN/ALTAR 84/3/CHEN/AEGILOPS SQUARROSA (TAUS)//BCN/4/MARCHOUCH-8/5/JOUDI | ISBW13-180-0TR-0TR-1TR |
| G-39 | 34450 | NEJMAH-12/Mace | ISBW14-479-19 |
| G-40 | 32708 | NESSER/SERI//TEVEE-1/SHUHA-6/3/AFIF | ISBW13-167-0TR-0TR-2TR |
| G-41 | 32705 | NESSER/SERI//TEVEE-1/SHUHA-6/3/FAKHER | ISBW13-166-0TR-0TR-2TR |
| G-42 | 33861 | Pirsabak 2008/AKRAM | ISBW13-477-020KUL-030DZ-3TR |
| G-43 | 35400 | PRL/2*PASTOR//SERI/4/MILAN/KAUZ//PRINIA/3/BABAX/5/HUBARA-3*2/SHUHA-4/6/KAMB2/PANDION | ISBW14T-10 -0SD-010KUL-3KUL-0KUL |
| G-44 | 35399 | PRL/2*PASTOR//SERI/4/MILAN/KAUZ//PRINIA/3/BABAX/5/HUBARA-3*2/SHUHA-4/6/KAMB2/PANDION | ISBW14T-10 -0SD-010KUL-2KUL-0KUL |
| G-45 | 35619 | QAFZAH-33/FLORKWA-2//Excalibur/3/DOUKKALA-33 | ISBW14T-205 -0SD-010KU-4KUL-0KUL |
| G-46 | 35620 | QAFZAH-33/FLORKWA-2//Excalibur/3/DOUKKALA-33 | ISBW14T-205 -0SD-010KU-5KUL-0KUL |
| G-47 | 33272 | REBWAH-12/5/ATTILA/3/URES/PRL//BAV92/4/WBLL1 | ICW 11-00386-0TR-0TR-3TR |
| G-48 | 32289 | REYNA-13/MASSIRA//SOONOT-10 | ICW 11-20006-1AP-0TR-2TR |
| G-49 | 32444 | REYNA-4//TRACHA-2/SHUHA-3/3/SOONOT-10 | ICW 11-20308-2AP-0TR-2TR |
| G-50 | 32311 | SERI.1B*2/3/KAUZ*2/BOW//KAUZ/4/ATTILA/HEILO/5/TAN//TEMPORALERA M 87/AGR/3/NG8319//SHA4/LIRA | ICW 11-20034-10AP-0TR-4TR |
| G-51 | 32592 | SOONOT-10/JELMOUD-1//SOONOT-7 | ICW 11-20831-7AP-0TR-3TR |
| G-52 | 35305 | SUNTOP/4/SERI.1B*2/3/KAUZ*2/BOW//KAUZ | ISBW14S-26 -0SD-010KUL-4KUL-0KUL |
| G-53 | 32747 | TEVEE-1/STAR'S'/3/ACHTAR*3//KANZ/KS85-8-4/4/SAKHA-93 | ISBW13-231-0TR-0TR-4TR |
| G-54 | 32628 | URES/BOW//OPATA/3/GIRWILL-7/4/AREEJ | ISBW13-040-0TR-0TR-5TR |
| G-55 | 32436 | VEE#8//JUP/BJY/3/F3.71/TRM/4/BCN/5/KAUZ/6/PASTOR/7/PASTOR/8/SOONOT-5/9/QAFZAH-33/FLORKWA-2 | ICW 11-20257-1AP-0TR-2TR |
| G-56 | 33305 | ATTILA*2/PBW65*2/5/KAUZ//ALTAR 84/AOS/3/MILAN/KAUZ/4/HUITES/6/WEEBILL-1/2*QAFZAH-21 | ICW 11-00568-0TR-0TR-3TR |
| G-57 | 34494 | HUBARA-15/ZEMAMRA-8//Mace | ISBW14-721-149 |
| G-58 | 33487 | HUBARA-16/2*SOMAMA-3//QAFZAH-33 | ICW 11-00920-0TR-0TR-3TR |
| G-59 | 32761 | HUBARA-3*2/SHUHA-4//CHAM-8 | ISBW13-266-0TR-0TR-1TR |
| G-60 | 32803 | HUBARA-3*2/SHUHA-4//KRASNOVODOPADSKAYA25/GRU-47 | ISBW13-286-0TR-0TR-5TR |
| G-61 | 33537 | HUBARA-3/SHUHA-4//AZAMAH-1 | ICW 11-01004-0TR-0TR-2TR |
| G-62 | 34230 | HUBARA-5/PASTOR-2/3/HOOSAM-8//CHAM-6/FLORKWA-2 | ICARC-WIP12063-10KUL-05DZ-3TR |
| G-63 | 32733 | KARAWAN-1/TALLO 3//JADIDA-2/3/TOROS | ISBW13-197-0TR-0TR-4TR |
| G-64 | 32859 | PASTOR-2/KATILA-13//HAMAM-5/3/9DEBEIRA | ISBW13-364-0TR-0TR-1TR |
| G-65 | 33859 | Pirsabak 2008/AKRAM | ISBW13-477-020KUL-030DZ-1TR |
| G-66 | 35617 | QAFZAH-33/FLORKWA-2//Excalibur/3/DOUKKALA-33 | ISBW14T-205 -0SD-010KU-2KUL-0KUL |
| G-67 | 33280 | QAMAR-2/3/RDWG/MILAN//MURGA | ICW 11-00443-0TR-0TR-2TR |
| G-68 | 33619 | SAAMID-3/OUASSOU-20 | ICW 11-01208-0TR-0TR-3TR |
| G-69 | 35681 | 02W50807/RSMF8 704//DOUMA 2 | ISBW14-73-0TR -0SD-010KUL-5MR-0MR |
| G-70 | 36010 | SHAMISS-5//HEILO/MIRIAM 41/3/ICARDA-SRRL-5 | ICW 11-20411-5AP -0SD -0SD-05KUL-2MR-0MR |
| G-71 | 35954 | SHARP/3/PRL/SARA//TSI/VEE#5/5/VEE/LIRA//BOW/3/BCN/4/KAUZ/6/MILAN/PASTOR/7/SUDAN#3/SHUHA-6 | ICW 11-20095-5AP -0SD -0SD-05KUL-1MR-0MR |
| G-72 | 35875 | SW89-5124*2/FASAN/3/CAZO/KAUZ//KAUZ/4/WBLL1/5/TNMU/CBRD//MILAN/SHA7 | ISBW13T-086-0TR -0SD-010KUL-1MR-0MR |
| G-73 | 35756 | 02W50807_1/4/BL2064//SW89.5124*2/FASAN/3/TILHI/5/HUBARA-3*2/SHUHA-4 | ISBW14-579-0TR -0SD-010KUL-2MR-0MR |
| G-74 | 36059 | SANDALL-5*2/CHAM-6 | ICW 11-20642-2AP -0SD -0SD-05KUL-6MR-0MR |
| G-75 | 35944 | TAZA-2/LAKTA-5//HAAMA-11/7/CADET/6/YUMAI13/5/NAI60/4/14.5/3/ODIN//CI13441/CANON | ISBW13-709-0TR -0SD -0SD-05KUL-5MR-0MR |
| G-76 | 35708 | 02W50807/4/PFAU/SERI.1B//AMAD/3/WAXWING/5/AGT-YOUNG | ISBW14-114-0TR -0SD-010KUL-5MR-0MR |
| G-77 | 35941 | HUBARA-3*2/SHUHA-4//AREEJ | ISBW13-298-0TR -0SD -0SD-05KUL-6MR-0MR |
| G-78 | 35816 | 02W50807_1/4/BL2064//SW89.5124*2/FASAN/3/TILHI/5/HUBARA-3*2/SHUHA-4 | ISBW14-779-0TR -0SD-010KUL-2MR-0MR |
| G-79 | 35877 | SW89-5124*2/FASAN/3/CAZO/KAUZ//KAUZ/4/WBLL1/5/TNMU/CBRD//MILAN/SHA7 | ISBW13T-086-0TR -0SD-010KUL-6MR-0MR |
| G-80 | 35679 | 02W50807/RSMF8 704//DOUMA 2 | ISBW14-73-0TR -0SD-010KUL-3MR-0MR |
| G-81 | 35850 | 05W90045/SOKOLL | ISBW14-833-0TR -0SD-010KUL-2MR-0MR |
| G-82 | 35901 | SHARP/3/PRL/SARA//TSI/VEE#5/5/VEE/LIRA//BOW/3/BCN/4/KAUZ/6/K7684.1/PRINIA | ICW 12-20246-0TR -0SD-010KUL-2MR-0MR |
| G-83 | 35748 | 02W50807/4/PFAU/SERI.1B//AMAD/3/WAXWING/5/GUARDIAN | ISBW14-498-0TR -0SD-010KUL-4MR-0MR |
| G-84 | 35923 | CHAM6/ATTILA//PASTOR/3/KAMB2/PANDION | ICW 12-20498-0TR -0SD-010KUL-4MR-0MR |
| G-85 | 35687 | 02W50807/RSMF8 704//HAMAM-4 | ISBW14-91-0TR -0SD-010KUL-3MR-0MR |
| G-86 | 35943 | HUBARA-3*2/SHUHA-4//HIDDAB/FLORKWA-2 | ISBW13-304-0TR -0SD -0SD-05KUL-4MR-0MR |
| G-87 | 35691 | 02W50807/RSMF8 704//HAMAM-4 | ISBW14-94-0TR -0SD-010KUL-1MR-0MR |
| G-88 | 35784 | 02W50807_1/4/BL2064//SW89.5124*2/FASAN/3/TILHI/5/REEHAB-2 | ISBW14-616-0TR -0SD-010KUL-3MR-0MR |
| G-89 | 35750 | 02W50807_1/RSMF8 704//REEHAB-2 | ISBW14-549-0TR -0SD-010KUL-4MR-0MR |
| G-90 | 35696 | 02W50807/4/BL2064//SW89.5124*2/FASAN/3/TILHI/5/DOUMA 2 | ISBW14-96-0TR -0SD-010KUL-8MR-0MR |
| G-91 | 35857 | HUBARA-3*2/SHUHA-4//REEVES | ISBW14-839-0TR -0SD-010KUL-5MR-0MR |
| G-92 | 35668 | CACHANILLA F2000*2/3/IRIHEA/BABAX//PASTOR/4/BURRION | ISBW14-51-0TR -0SD-010KUL-3MR-0MR |
| G-93 | 35966 | AGUILAL/FLAG-3/5/CNO79//PF70354/MUS/3/PASTOR/4/CROC-1/AE.SQUARROSA (224)//OPATA/6/MILAN/KAUZ//PASTOR/3/PASTOR | ICW 11-20209-3AP -0SD -0SD-05KUL-3MR-0MR |
| G-94 | 35879 | SW89-5124*2/FASAN/3/CAZO/KAUZ//KAUZ/4/WBLL1/5/TNMU/CBRD//MILAN/SHA7 | ISBW13T-230-0TR -0SD-010KUL-2MR-0MR |
| G-95 | 35938 | HUBARA-3*2/SHUHA-4//AREEJ | ISBW13-298-0TR -0SD -0SD-05KUL-1MR-0MR |
| G-96 | 35813 | 02W50807_1/4/BL2064//SW89.5124*2/FASAN/3/TILHI/5/HUBARA-3*2/SHUHA-4 | ISBW14-778-0TR -0SD-010KUL-5MR-0MR |
| G-97 | 35865 | SERI.1B*2/3/KAUZ*2/BOW//KAUZ/4/02W50807/RSMF8 704 | ISBW14-904-0TR -0SD-010KUL-3MR-0MR |
| G-98 | 35706 | 02W50807/4/OASIS/SKAUZ//4*BCN/3/2*PASTOR/5/HUBARA-3*2/SHUHA-4 | ISBW14-111-0TR -0SD-010KUL-2MR-0MR |
| G-99 | 35794 | 02W50807_1/4/BL2064//SW89.5124*2/FASAN/3/TILHI/5/DOUMA 2 | ISBW14-733-0TR -0SD-010KUL-3MR-0MR |
| G-100 | 35918 | MILAN/KAUZ//PASTOR/3/PASTOR/4/K7684.1/PRINIA | ICW 12-20474-0TR -0SD-010KUL-2MR-0MR |
| G-101 | 35799 | 02W50807_1/4/BL2064//SW89.5124*2/FASAN/3/TILHI/5/BOBICHO | ISBW14-743-0TR -0SD-010KUL-1MR-0MR |
| G-102 | 35886 | FRAME//MILAN/KAUZ/3/PASTOR/7/TILHI/5/PF74354//LD/ALD/4/2*BR12*2/3/JUP//PAR214*6/FB6631/6/ATTILA/2*PASTOR | ICW 12-20165-0TR -0SD-010KUL-3MR-0MR |
| G-103 | 35744 | 02W50807/4/PFAU/SERI.1B//AMAD/3/WAXWING/5/ADEL-6 | ISBW14-474-0TR -0SD-010KUL-4MR-0MR |
| G-104 | 35935 | HUBARA-3*2/SHUHA-4//BERKUME | ISBW13-288-0TR -0SD -0SD-05KUL-2MR-0MR |
| G-105 | 35973 | SANDALL-3/4/PASTOR//HXL7573/2*BAU/3/HXL7573/2*BAU/5 /WORRAKATTA/2*PASTOR | ICW 11-20219-2AP -0SD -0SD-05KUL-2MR-0MR |
| G-106 | 35841 | BABAX/LR42//BABAX/3/BAVIACORA/4/HEILO/5/SOKOLL | ISBW14-811-0TR -0SD-010KUL-7MR-0MR |
| G-107 | 35871 | MILAN/S87230//BABAX/3/02W50807/RSMF8 704 | ISBW14-1023-0TR -0SD-010KUL-1MR-0MR |
| G-108 | 35818 | 02W50807_1/4/BL2064//SW89.5124*2/FASAN/3/TILHI/5/HUBARA-3*2/SHUHA-4 | ISBW14-779-0TR -0SD-010KUL-5MR-0MR |
| G-109 | 35827 | 02W50807_1/4/PFAU/SERI.1B//AMAD/3/WAXWING/5/HUBARA-3*2/SHUHA-4 | ISBW14-795-0TR -0SD-010KUL-2MR-0MR |
| G-110 | 35694 | 02W50807/RSMF8 704/4/ATENA-1/GAMDOW-3/3/MON'S'/ALD'S'//ALDAN'S'/IAS58 | ISBW14-96-0TR -0SD-010KUL-3MR-0MR |
| G-111 | 36039 | PFAU/MILAN/5/CHEN/AEGILOPS SQUARROSA (TAUS)//BCN/3/VEE#7/BOW/4/PASTOR/6/REYNA-12/7/K7684.1/PRINIA | ICW 11-20498-4AP -0SD -0SD-05KUL-5MR-0MR |
| G-112 | 35736 | 02W50807/4/PFAU/SERI.1B//AMAD/3/WAXWING/5/EGA-WENTWORTH | ISBW14-380-0TR -0SD-010KUL-2MR-0MR |
| G-113 | 35742 | 02W50807/4/PFAU/SERI.1B//AMAD/3/WAXWING/5/ADEL-6 | ISBW14-460-0TR -0SD-010KUL-2MR-0MR |
| G-114 | 35659 | PBW343*2/KHVAKI//BAVIACORA*2/3/HEILO/4/HUBARA-3*2/SHUHA-4 | ISBW14-36-0TR -0SD-010KUL-2MR-0MR |
| G-115 | 35919 | MILAN/KAUZ//PASTOR/3/PASTOR/4/K7684.1/PRINIA | ICW 12-20474-0TR -0SD-010KUL-3MR-0MR |
| G-116 | 35718 | 02W50807/4/PFAU/SERI.1B//AMAD/3/WAXWING/5/HUBARA-3*2/SHUHA-4 | ISBW14-175-0TR -0SD-010KUL-1MR-0MR |
| G-117 | 35725 | 02W50807/4/PFAU/SERI.1B//AMAD/3/WAXWING/5/ATENA-1/GAMDOW-3/3/MON'S'/ALD'S'//ALDAN'S'/IAS58 | ISBW14-229-0TR -0SD-010KUL-2MR-0MR |
| G-118 | 35958 | VEE#8//JUP/BJY/3/F3.71/TRM/4/BCN/5/KAUZ/6/PASTOR/7/PASTOR/8/SHAMISS-3/9/CROW'S'/BOW'S' -3-1994/95//TEVEE'S'/TADINIA | ICW 11-20140-1AP -0SD -0SD-05KUL-1MR-0MR |
| G-124 | 36248 | SOONOT-10/HUBARA-15/3/MILAN/S87230//BABAX | ICW 11-20311-4AP-040KUL-020KUL-3MR-0MR-0KUL |
| G-127 | 36116 | ATTILA*2/PBW65*2//MURGA/3/HUBARA-3/SHUHA-4 | ICW 11-00652-0TR -0SD -0SD-05KUL-7MR-0MR |
| G-128 | 36120 | WAXWING*2/HEILO//HUBARA-3*2/SHUHA-4 | ICW 11-00670-0TR -0SD -0SD-05KUL-2MR-0MR |
| G-129 | 36129 | UP2338*2/KKTS*2//YANAC/3/HUBARA-16/2*SOMAMA-3 | ICW 11-00733-0TR -0SD -0SD-05KUL-1MR-0MR |
| G-130 | 36137 | KBIRD/5/KAUZ//ALTAR 84/AOS/3/MILAN/KAUZ/4/HUITES/6/HUBARA-3/SHUHA-4 | ICW 11-00778-0TR -0SD -0SD-05KUL-1MR-0MR |
| G-131 | 36138 | KBIRD/5/KAUZ//ALTAR 84/AOS/3/MILAN/KAUZ/4/HUITES/6/HUBARA-3/SHUHA-4 | ICW 11-00778-0TR -0SD -0SD-05KUL-2MR-0MR |
| G-132 | 36139 | KBIRD/5/KAUZ//ALTAR 84/AOS/3/MILAN/KAUZ/4/HUITES/6/HUBARA-3/SHUHA-4 | ICW 11-00778-0TR -0SD -0SD-05KUL-3MR-0MR |
| G-133 | 36148 | NAC/TH.AC//3*PVN/3/MIRLO/BUC/4/2*PASTOR/5/KAUZ//ALTAR 84/AOS/3/MILAN/KAUZ/4/HUITES/6/KAUZ//ALTAR 84/AOS/3/MILAN/KAUZ/4/HUITES/7/HUBARA-3*2/SHUHA-4 | ICW 11-00802-0TR -0SD -0SD-05KUL-4MR-0MR |
| G-134 | 36174 | MOUKA-4/RAYON//SIDS12 | ICWIP12-030 -029SD -0SD -0SD-05KUL-4MR-0MR |
| G-135 | 36180 | HUBARA-2/QAFZAH-21//DOVIN-2/6/KADAR-1/4/VAN'S'/3/CNDR'S'/ANA//CNDR'S'/MUS'S'/5/SOMAMA-3 | ICWIP12-084 -034SD -0SD -0SD-05KUL-5MR-0MR |
| G-136 | 36187 | USHER-18/5/DEBEIRA/4/KAUZ//ALTAR 84/AOS/3/KAUZ | ICWIP13-45 -0SD-050KUL-020KUL-6MR-0MR |
| G-137 | 36193 | KAUZ'S'/SERI/4/SERI.1B*2/3/KAUZ*2/BOW//KAUZ/6/ATRIS-10/4/PASTOR /3/KAUZ*2/ OPATA//KAUZ/5/CHAM-6/TUI'S' | ICWIP13-73 -0SD-050KUL-020KUL-4MR-0MR |
| G-138 | 36212 | HAMAM-2/DEEK-2//GIZA-168/3/Axe | ICWIP13-255 -0SD-050KUL-020KUL-3MR-0MR |
| G-139 | 36214 | HUBARA-16/2*SOMAMA-3/4/HUBARA-8/3/MON'S'/ALD'S'//BOW'S'/5/ATTILA-7/KATILA-12 | ICWIP13-298 -0SD-050KUL-020KUL-4MR-0MR |
| G-140 | 36216 | BOW #1/FENGKANG 15//NESMA*2/261-9/3/DUCULA/4/SAKHA-94/5/GEMMIZA-12 | ICWIP13-313 -0SD-050KUL-020KUL-5MR-0MR |
| G-141 | 36248 | SOONOT-10/HUBARA-15/3/MILAN/S87230//BABAX | ICW 11-20311-4AP-040KUL-020KUL-3MR-0MR |
| G-143 | 36300 | HUBARA-3*2/SHUHA-4//HUBARA-3*2/SHUHA-4 | ISBW13-309-0TR-040KUL-020KUL-1MR-0MR |
| G-144 | 36302 | HUBARA-3*2/SHUHA-4//HUBARA-3*2/SHUHA-4 | ISBW13-309-0TR-040KUL-020KUL-3MR-0MR |
| G-145 | 36308 | HUBARA-3*2/SHUHA-4//NARC 2011 | ISBW13-427-0TR-040KUL-020KUL-3MR-0MR |
| G-146 | 36310 | HUBARA-3*2/SHUHA-4//NARC 2011 | ISBW13-427-0TR-040KUL-020KUL-5MR-0MR |
| G-147 | 36311 | HUBARA-3*2/SHUHA-4//NARC 2011 | ISBW13-427-0TR-040KUL-020KUL-6MR-0MR |
| G-148 | 36322 | Pirsabak 2008/BASHIR | ISBW13-474-0TR-040KUL-020KUL-5MR-0MR |
| G-149 | 36326 | Saleem-2000/BASHIR | ISBW13-505-0TR-040KUL-020KUL-1MR-0MR |
| G-150 | 36350 | GEMMIZA-12/SIDS-12 | ICARC-WIP13385-0TR-040KUL-020KUL-4MR-0MR |
| G-151 | 36372 | CAL/NH//H567.71/3/SERI/4/CAL/NH//H567.71/5/2*KAUZ/6/PASTOR/7/YANAC/8/CAL/NH//H567.71/3/SERI/4/CAL/NH//H567.71/5/2*KAUZ/6/PASTOR/9/SANDALL-3 | ICW 11-00601-0TR-040KUL-020KUL-5MR-0MR |
| G-152 | 36379 | KBIRD/5/KAUZ//ALTAR 84/AOS/3/MILAN/KAUZ/4/HUITES/6/HUBARA-3/SHUHA-4 | ICW 11-00778-0TR-040KUL-020KUL-4MR-0MR |
| G-153 | 36380 | KBIRD/5/KAUZ//ALTAR 84/AOS/3/MILAN/KAUZ/4/HUITES/6/HUBARA-3/SHUHA-4 | ICW 11-00778-0TR-040KUL-020KUL-7MR-0MR |
| G-154 | 36382 | PBW343*2/KUKUNA/3/PGO/SERI//BAV92/4/NABUQ-6 | ICW 11-00786-0TR-040KUL-020KUL-6MR-0MR |
| G-155 | 36387 | C80.1/3*BATAVIA//2*WBLL1/3/2*FRET2/TUKURU//FRET2/4/MILAN/S87230//BABAX | ICW 11-00798-0TR-040KUL-020KUL-1MR-0MR |
| G-156 | 36392 | NAC/TH.AC//3*PVN/3/MIRLO/BUC/4/2*PASTOR/5/KAUZ//ALTAR 84/AOS/3/MILAN/KAUZ/4/HUITES/6/KAUZ//ALTAR 84/AOS/3/MILAN/KAUZ/4/HUITES/7/HUBARA-3*2/SHUHA-4 | ICW 11-00802-0TR-040KUL-020KUL-6MR-0MR |
| G-157 | 36396 | SUNCO/2*PASTOR/3/WHEAR/KUKUNA//WHEAR | ICW 11-00860-0TR-040KUL-020KUL-2MR-0MR |
| G-158 | 36401 | HUBARA-3*2/SHUHA-4//LALOUB-2 | ICW 11-01026-0TR-040KUL-020KUL-4MR-0MR |
| G-159 | 36404 | HUBARA-3*2/SHUHA-4//SHAMISS-4 | ICW 11-01028-0TR-040KUL-020KUL-2MR-0MR |
| G-160 | 36406 | Millenium/RUTH-2 | ICW 11-01105-0TR-040KUL-020KUL-6MR-0MR |
| G-161 | 36429 | CHAM-6/AGUILAL | ICW-TR 10-00249-1AP-0TR-0MR-5MR-0MR |
| G-162 | 36451 | ATTILA-7/KBG-01 | ICW-TR 10-00263-7AP-0TR-0MR-3MR-0MR |
| G-163 | 36509 | SANDALL-3/GOUMRIA-3 | ICW-TR 10-00388-1AP-0TR-0MR-3MR-0MR |
| G-164 | 36537 | BJY/COC//PRL/BOW/3/JAWAHIR-19/SAMIRA-7 | ICW-TR 10-20059-5AP-1AP-0TR-0MR-3MR-0MR |
| G-165 | 36551 | YAR/AE.SQUARROSA (783)/4/GOV/AZ//MUS/3/SARA/5/MYNA/VUL//JUN/6/REYNA-16 | ICW-TR 10-00238-1AP-0TR-0MR-3MR-0MR |
| G-166 | 36623 | WBLL1*2/KURUKU//HEILO/3/WBLL1*2/KURUKU/4/HUBARA-3*2/SHUHA-4 | ICW 11-00673-0TR-0MR-4MR-0MR |
| G-167 | 36624 | WBLL1*2/KURUKU//HEILO/3/WBLL1*2/KURUKU/4/HUBARA-3*2/SHUHA-4 | ICW 11-00673-0TR-0MR-5MR-0MR |
| G-168 | 36642 | KBIRD/5/KAUZ//ALTAR 84/AOS/3/MILAN/KAUZ/4/HUITES/6/PRL/2*PASTOR/4/CHOIX/STAR/3/HE1/3*CNO79//2*SERI | ICW 11-00774-0TR-0MR-3MR-0MR |
| G-169 | 36650 | KBIRD//INQALAB 91*2/TUKURU/3/HUBARA-3/SHUHA-4 | ICW 11-00781-0TR-0MR-5MR-0MR |
| G-170 | 36653 | PBW343*2/KUKUNA/3/PGO/SERI//BAV92/4/HUBARA-3/SHUHA-4 | ICW 11-00785-0TR-0MR-5MR-0MR |
| G-171 | 36746 | ATTILA-7 //HUBARA-3*2/SHUHA-4-1 | ISBW13-030-0TR-0MR-3MR-0MR |
| G-172 | 36748 | ATTILA-7 //HUBARA-3*2/SHUHA-4 | ISBW13-031-0TR-0MR-1MR-0MR |
| G-173 | 36778 | SHUHA-4/FLORKWA-4//HUBARA-3/6/88ZHONG218//CTK/VEE/3/KVZ/GV//PR/4/KRASNOVODOPADSKAYA25/5/KS82117/MLT | ISBW13-405-0TR-0MR-3MR-0MR |
| G-174 | 36791 | KARAWAN-1/TALLO 3//REGRAG-1/3/OUASSOU-11 | ISBW13-674-0TR-0MR-7MR-0MR |
| G-176 | 36825 | QAFZAH-25/ANGI-1//HAIEL-1/4/VEE/PJN//2*TUI/3/WH576 | ICARC-WIP13190-0TR-0MR-3MR-0MR |
| G-177 | 36846 | MILAN/KAUZ//PRINIA/3/BABAX/4/PASTOR/3/KAUZ*2/OPATA//KAUZ/5/REYNA-17 | ICW 11-20106-3AP-0MR-4MR-0MR |
| G-178 | 37432 | CHAM-6/PERW//MILAN/PASTOR/3/CHAM-6/PERW/4/NADER | ISBW13-439-0TR -0SD -0SD-2SD -0SD |
| G-179 | 37838 | SERI.1B//KAUZ/HEVO/3/AMAD/4/HXL8246/KAUZ/6/REYNA-4//KAMB2/PANDION/5/PRL/2*PASTOR//SERI/4/MILAN/KAUZ//PRINIA/3/BABAX | ISBW15TC-TR-246 -0SD-010KUL-5KUL |
| G-180 | 37848 | 4WON-IR-257/5/YMH/HYS//HYS/TUR3055/3/DGA /4/ VPM / MOS/7/BABAX/LR42//BABAX/3/BAVIACORA/4/HEILO/5/LAKTA-1/QAFZAH-21/6/HUBARA-5/ANGI-1 | ISBW15TC-TR-344 -0SD-010KUL-5KUL |
| G-181 | 37842 | Guna = SERI.1B//KAUZ/HEVO/3/AMAD/4/KAUZ/GYS//KAUZ/7/PASTOR/3/GEN*2//BUC/FLK/4/PASTOR/5/KAMB2/PANDION/6/02W50807/RSMF8 704 | ISBW15TC-TR-261 -0SD-010KUL-4KUL |
| G-182 | 37918 | SERI.1B//KAUZ/HEVO/3/AMAD/4/KAUZ/GYS//KAUZ/5/RIL 148 | ISBW15-TR-1128-0SD-010KUL-2KUL |
| G-183 | 37889 | HEILO/3/OASIS/SKAUZ//4*BCN/4/Av 36-29-34#20 | ISBW15-TR-674 -0SD-010KUL-3KUL |
| G-184 | 37904 | 02W50807_1/4/PFAU/SERI.1B//AMAD/3/WAXWING/5/Av 36-29-34#20 | ISBW15-TR-909 -0SD-010KUL-KUL-3KUL |
| G-185 | 37946 | CACHANILLA F2000*2/3/IRIHEA/BABAX//PASTOR/4/DORADE-5//KS82117/MLT | ISBW15-SxW-TR-250 -0SD-010KUL-3KUL |
| G-186 | 37921 | SERI.1B//KAUZ/HEVO/3/AMAD/4/KAUZ/GYS//KAUZ/5/RIL 148 | ISBW15-TR-1128-0SD-010KUL-5KUL |
| G-187 | 37991 | KAMB2/PANDION//Av Yr 15 | ISBW15-TR-368 -0SD-010KUL-2KUL |
| G-188 | 37769 | HEILO/MIRIAM 41/6/VEE/PJN//2*TUI/3/2*MILAN/KAUZ/4/ATTILA/HEILO/5/BUMPER | ISBW15TC-TR-16 -0SD-010KUL-5KUL |
| G-189 | 37956 | CROC_1/AE.SQUARROSA (205)//KAUZ/3/2*KAUZ*2/YACO//KAUZ/4/ERYT783-96/SHARK-1 | ISBW15-SxW-TR-391 -0SD-010KUL-5KUL |
| G-190 | 37925 | HEILO/3/OASIS/SKAUZ//4*BCN/6/4WON-IR-257/5/YMH/HYS//HYS/TUR3055/3/DGA /4/ VPM / MOS /7/SERI.1B//KAUZ/HEVO/3/AMAD/4/KAUZ/GYS//KAUZ/5/02W50807/4/PFAU/SERI.1B//AMAD/3/WAXWING | ISBW15 T-TR-144 -0SD-010KUL-4KUL |
| G-191 | 37752 | ATRAK/5/KAUZ/3/MYNA/VUL//BUC/FLK/4/MILAN/7/PASTOR/3/GEN*2//BUC/FLK/4/PASTOR/5/CROC_1/AE.SQUARROSA (205)//KAUZ/3/2*KAUZ*2/YACO//KAUZ/6/02W50807/RSMF8 704 | ISBW15TC-TR-03 -0SD-010KUL-3KUL |
| G-192 | 37804 | HEILO/3/SW89.5277/BORL95//SKAUZ/7/PASTOR/3/GEN*2//BUC/FLK/4/PASTOR/5/CROC_1/AE.SQUARROSA (205)//KAUZ/3/2*KAUZ*2/YACO//KAUZ/6/02W50807/RSMF8 704 | ISBW15TC-TR-160 -0SD-010KUL-1KUL |
| G-193 | 37821 | 02W50807/RSMF8 704/7/ATTILA/4/WEAVER/TSC//WEAVER/3/WEAVER/5/ ATTILA/2*PASTOR//FISCAL/6/DOUKKALA-34 | ISBW15TC-TR-189 -0SD-010KUL-5KUL |
| G-194 | 37840 | Guna = SERI.1B//KAUZ/HEVO/3/AMAD/4/KAUZ/GYS//KAUZ/7/PASTOR/3/GEN*2//BUC/FLK/4/PASTOR/5/KAMB2/PANDION/6/02W50807/RSMF8 704 | ISBW15TC-TR-261 -0SD-010KUL-2KUL |
| G-195 | 37934 | TEMERIND-5/Solh/7/HEILO/3/SW89.5277/BORL95//SKAUZ/6/02W50807/5/PSN/BOW//SERI/3/MILAN/4/ATTILA | ISBW15 T-TR-154 -0SD-010KUL-1KUL |
| G-196 | 35954 | SHARP/3/PRL/SARA//TSI/VEE#5/5/VEE/LIRA//BOW/3/BCN/4/KAUZ/6/MILAN/PASTOR/7/SUDAN#3/SHUHA-6 | ICW 11-20095-5AP -0SD -0SD-05KUL-1MR-0MR |
